# Which specific modes of exercise training are most effective for breast related cancer fatigue? Network meta-analysis

**DOI:** 10.3389/fonc.2025.1491634

**Published:** 2025-02-26

**Authors:** Ying Li, Jianhua Zhang, Di Hu, Lei Gao, Ting Huang

**Affiliations:** ^1^ College of Sports Science, Jishou University, Jishou, Hunan, China; ^2^ School of Physical Education and Arts, Hunan University of Medicine, Huaihua, Hunan, China; ^3^ Department of Neurology, Zhongshan Hospital Affiliated to Dalian University, Dalian, Liaoning, China; ^4^ School of Nursing, Dalian University, Dalian, Liaoning, China; ^5^ Early Intervention Ward, The Third People’s Hospital of Ganzhou, Ganzhou, Jiangxi, China

**Keywords:** breast cancer, CFR, network meta-analysis, meta - analysis, cancer

## Abstract

**Objective:**

The objective of this study was to examine the impact of various exercise modalities on Cancer-Related Fatigue (CRF) among breast cancer patients.

**Methods:**

A computerized search was conducted on databases including PubMed, Embase, Cochrane Library, Web of Science, CNKI, VIP, and Wanfang Database up to October 2023. Selection criteria were applied to include or exclude studies, resulting in the inclusion of 65 articles for comparison of the effects of 12 distinct exercise interventions on individuals with breast cancer.

**Results:**

The 65 studies used 12 different measures of exercise. Network meta results show that Compared with Other exercise (OE), Baduanjin exercise (BE), Qigong (QG), Control group (CG), Tai Chi (TC) improved significantly in CFR. The effect of Yoga (YG) on improving sleep quality is better than Control group (CG) and Baduanjin exercise (BE). Compared with Control group (CG), Tai Chi (TC) and Yoga (YG) are more beneficial to improve the quality of life of breast cancer patients. Tai Chi (TC) is better than Multimodal exercise (ME), Other exercise (OE), Baduanjin exercise (BE), Pilates exercise (PE), Yoga (YG), Qigong (QG), Dance exercise (DE), Qigong (QG) in improving depression in breast cancer patients.

**Conclusion:**

The study revealed that Tai Chi demonstrates positive effects in ameliorating CRF, enhancing quality of life, and alleviating depressive symptoms among breast cancer patients. Moreover, yoga exhibits favorable effects in improving sleep quality in this patient group. Nevertheless, additional randomized controlled trials (RCTs) are warranted in the future to delve deeper into the effectiveness and underlying mechanisms of these exercise interventions.

## Introduction

Breast cancer is the most common cancer among women. According to the American Cancer Society, the incidence of female breast cancer has been rising annually by 0.5%, with approximately 287,850 new cases diagnosed every year in the United States, accounting for 31% of all new cancer diagnoses in women (1). In recent years, the introduction of neoadjuvant therapy has significantly improved survival rates for patients. However, survivors often face various physical and psychological challenges, including premature menopause, body image issues, fatigue, and depression (2–4). Cancer-related fatigue (CRF) is a frequent symptom experienced by patients with breast cancer (5). It is defined as a distressing and persistent subjective sensation of physical, emotional, or cognitive exhaustion associated with cancer or its treatment (6).

Unlike normal tiredness, CRF is severe, prolonged, unresponsive to rest or sleep, and interferes with daily activities, thereby significantly diminishing patients’ overall quality of life (7–11).

Exercise is widely recognized as an effective non-pharmacological intervention for patients with cancer (12–14). A growing body of evidence supports the significant benefits of increased physical activity in improving psychological outcomes and physical health as well as reducing fatigue in these patients (14–18). Yoga (YG) is a form of physical and mental exercise that integrates the mind and body through postures, pranayama, and meditation. This approach can alleviate patients’ discomfort by addressing both physical and cognitive challenges (19). Resistance training (RT) can reduce plasma and tissue-specific inflammation, inhibit nerve signal transmission, and mitigate symptoms of fatigue (20, 21). Traditional meta-analyses have also demonstrated the efficacy of YG and RT in reducing CRF in patients with breast cancer (22, 23). In a comprehensive overview of rehabilitation interventions, Olsson et al. found a positive impact of YG on CRF (24). Additionally, Zou’s study revealed that patients with breast cancer who engaged in aerobic exercise exhibited significantly lower Revised Piper Fatigue Scale (RPFS) scores compared to those receiving usual care, indicating the potential of aerobic exercise to alleviate CRF in patients undergoing chemotherapy (25). However, another systematic review showed that while yoga was more effective than aerobic exercise in improving CRF, aerobic exercise and tai chi did not significantly affect CRF (26). These findings, although validating the effectiveness of exercise for CRF in patients with breast cancer, remain controversial. The occurrence of CRF can significantly impair the health-related quality of life in breast cancer survivors. Practical exercise training has been shown to enhance mitochondrial function and plasticity, thereby mitigating the occurrence of CRF (27–29). Therefore, to effectively alleviate fatigue in patients with breast cancer, it is crucial to determine the most appropriate and optimal exercise modalities from various training methods.

Network meta-analysis (NMA) is a sophisticated approach to meta-analysis that enables the evaluation of multiple treatment comparisons (30). This method facilitates the calculation and comparison of treatment estimates from both direct and indirect evidence using a common comparator against which various interventions can be assessed. Consequently, NMA allows for the assessment of the comparative effectiveness of diverse interventions, even in cases that have not been directly compared (31). Although two previous NMA studies have been conducted (32, 33), they were limited to examining the effects of various exercise interventions, without exploring other intervention types. Thus, the present study aims to perform an NMA using relevant randomized controlled trials to compare the efficacy of diverse interventions on CRF in patients with breast cancer. The results of this study will be crucial in formulating clinical practice guidelines that recommend the most effective intervention for improving CRF outcomes in this patient population.

## Methods

This NMA was designed based on the guidelines for Preferred Reporting Items of Systematic Review and Network Meta-Analysis (34), which are registered in the PROSPERO database (CRD42024525453).

### Search strategies

A systematic search for randomized controlled trials (RCTs) focusing on cancer-related fatigue (CRF) in breast cancer patients, up to October 2023, was carried out across multiple databases including PubMed, Web of Science, Embase, Cochrane Library, China National Knowledge Infrastructure (CNKI), and Wanfang. The search strategy utilized a combination of Mesh Terms and free-text terms. Detailed information regarding the search strategy can be accessed in **Supplementary Material Appendix 1**.

### Study selection

For the purpose of this study, independent reviewers YL and LG were chosen to screen the titles and abstracts of the retrieved literature using predefined search strategies in order to identify relevant studies meeting the inclusion criteria. In the event of any disagreements, TH conducted checks and facilitated discussions to reach a consensus. Duplicate records were removed using EndNote software (35)to ensure data integrity. Subsequently, a thorough assessment of the full-text articles of potentially eligible studies was carried out based on predefined inclusion and exclusion criteria. Any discrepancies between the reviewers were resolved through discussion, with the aid of EndNote software for managing this phase.

### Inclusion criteria

The included studies were required to meet the following criteria: (1) Study type: randomized controlled trials (RCTs). (2) Inclusion of adult patients (18 years or older) diagnosed with breast cancer, without restriction on cancer stage or current treatment options. (3) Interventions: Walking Exercise (WE), Home Exercise (HE), Resistance Training (RT), Aerobic Exercise (AE), Yoga (YG), Stretching Exercise (STE), Music Interventions (MI), Relaxation Training (RT), Baduanjin Exercise (BE), Auriculotherapy (AT), Water-based Exercise (WBE), Pilates Exercise (PE), Cognitive Behavioral Therapy (CBT), Dance Exercise (DE), Tai Chi (TC), and Qigong. (4) Outcomes: inclusion of at least one outcome measure. The primary outcome measure for this study was cancer-related fatigue (CRF), assessed using various scales including the Functional Assessment of Cancer Therapy (FACT)-Fatigue Scale, European Organization for Research and Treatment of Cancer Quality of Life Questionnaire (EORTC QLQC30), Piper Fatigue Scale (PFS), Schwartz Cancer Fatigue Scale (SCFS), and Multidimensional Fatigue Inventory (36).Secondary outcomes included sleep quality measured by the Pittsburgh Sleep Quality Index (PSQI), quality of life assessed by the Breast Cancer Therapeutic Function Assessment (FACT-B), and self-rated depression measured by the Self-Rating Depression Scale (SDS), Hospital Anxiety and Depression Scale (HADS), Beck Depression Inventory (BDI), and Center for Epidemiologic Studies of Depression (CES-D). Additional details regarding each intervention can be found in **Supplementary Material Appendix 2**, and information about each outcome measure is provided in **Supplementary Material Appendix 3**.

### Exclusion criteria

(1) Patients presenting with severe complications. (2) Studies reporting outcomes that did not align with the design of the current study. (3) Studies providing data that could not be integrated due to incorrect or incomplete information. (4) non-English and non-Chinese studies.

### Data extraction

The reviewers independently extracted the following data from the included studies: first author, publication year, country of origin, sample size, body mass index (BMI), age, weight, height, intervention details, tumor stage, intervention duration, intervention frequency, and outcome measures. Data were reported as mean ± standard deviation.

### Risk of bias assessment

Two reviewers (LG and YL) independently evaluated the risk of bias, and any discrepancies were resolved by a third reviewer using Cochrane collaboration tools. These tools assessed various factors including sequence generation, allocation concealment, blinding, incomplete outcome data, selective outcome reporting, and other sources of bias (37). Each criterion was categorized as having a low, unclear, or high risk of bias (38).

### Data analysis

For NMA, the “Netmeta” package (39)in R-4.2.1 software (40)was utilized. Network plots were generated using the “network plot” feature in STATA 15.1 to visually represent different interventions and their favorable comparisons. Nodes represented various interventions, while edges depicted the favorable intervention relationships. Inconsistencies between direct and indirect comparisons were assessed using the node-splitting method (41). Random effects network meta-analysis was conducted to estimate combined effects and 95% confidence intervals (95% CI). When analyzing results or evaluating standardized mean differences (SMD), the mean difference (MD) was considered as the treatment effect for studies with the same unit of measurement. Pairwise random effects meta-analysis was performed to compare different exercise treatments. The *I* ^2^ statistic was used to evaluate heterogeneity across all pairwise comparisons, and publication bias was assessed using Egger’s test p-value. Funnel plots were employed to identify publication bias and secondary study effects, based on the results from multiple reported studies.

## Results

### Literature selection

**Figure 1:**
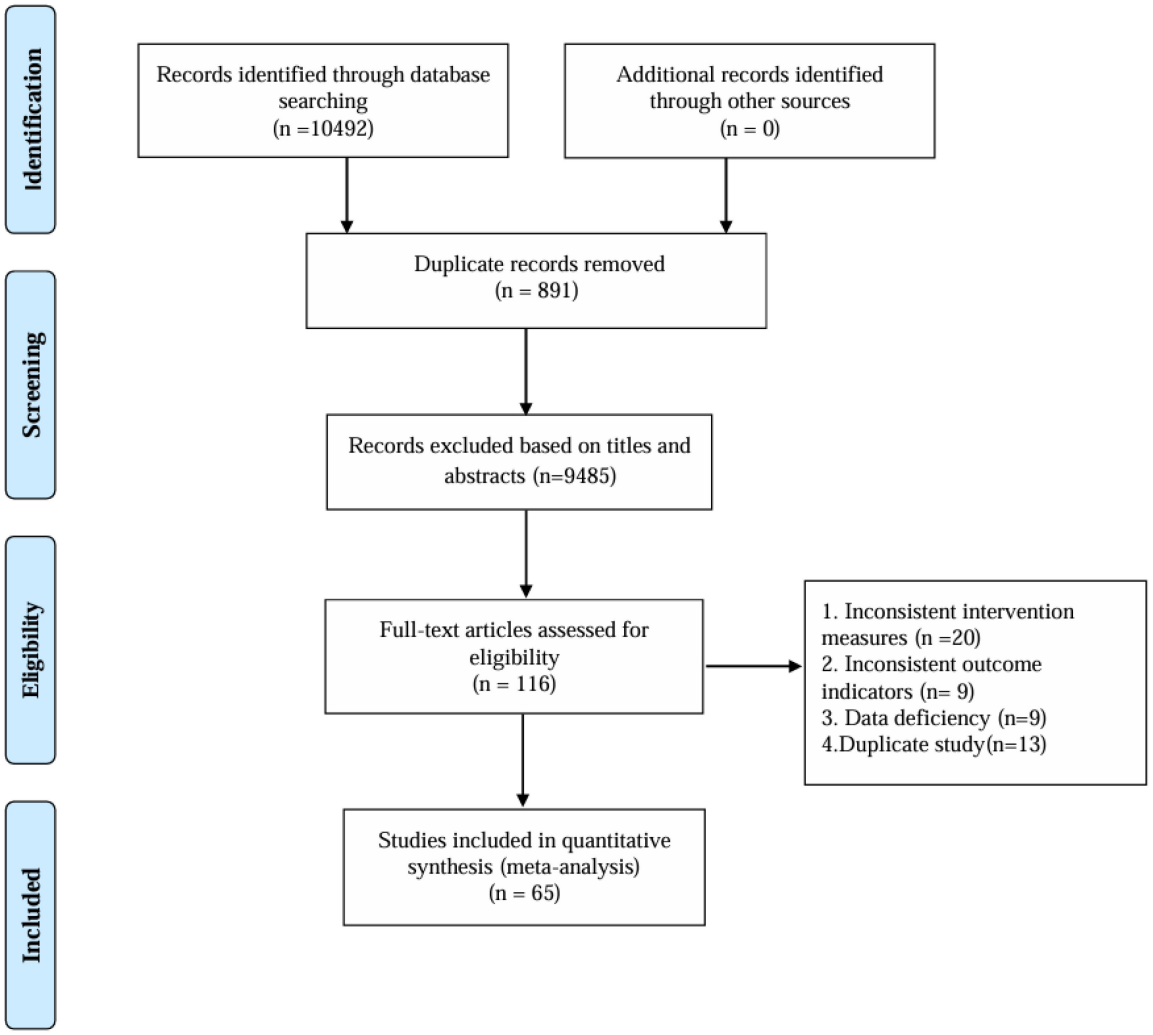
Flow of trials through the review.

After removing duplicates, 10492 records were retrieved, and 10376 papers were discarded. The full text of the remaining 116 records was analyzed, and 52 cases did not satisfy the inclusion criteria: inconsistent intervention measures (20), inconsistent outcome indicators (9), data deficiency (9), and duplicate study (13). In the end, 65 (39–103) studies were included. **Figure 1** shows the research flowchart.

### Study and participant characteristics

**Figure 2:**
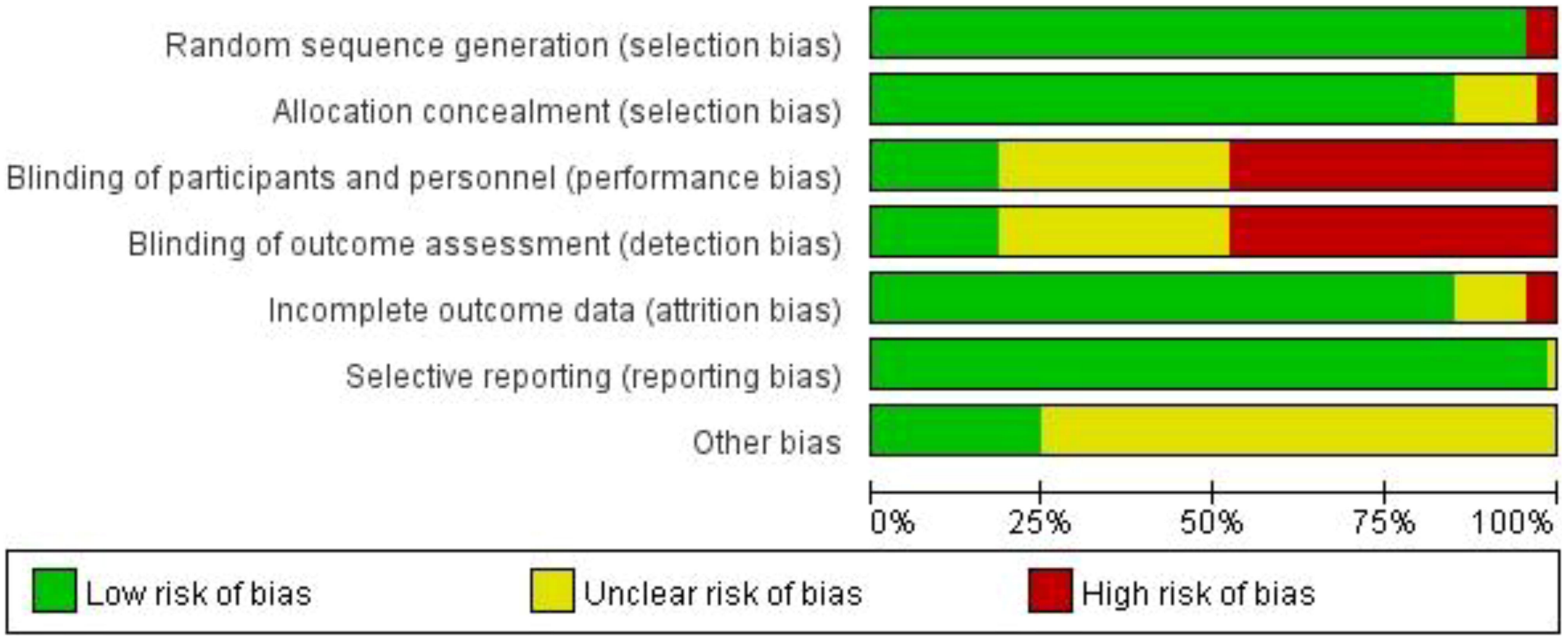
Percentage of studies examining the efficacy of interventions in patients with breast cancer with low, unclear, and high risk of bias for each feature of the Cochrane Risk of Bias Tool.

Studies comparing the effects of 12 various interventions on patients with breast cancer, published between 2001 and 2022, were included. The intervention durations ranged from 1 week to 12 months, and a total of 5,300 patients were reported in the included studies. Among these studies, 48 reported CFR, 16 reported PSQI, 14 reported FACT-B and 18 reported depression. The participants had an average age of 18-69 years, an average BMI of 22.05 ± 2.67-29.27 ± 5.92, an average height of 140.2 ± 23.07-170.2 ± 5.4 cm, and an average weight of 50.4 ± 7.4-74.3 ± 17.0 Kg. **Table 1** shows the characteristics of the studies and participants. The risk of bias assessment for each study is presented in **Additional Document 1 (Appendix 4)**, and **Figure 2** presents the aggregated data.

**Table 1:** General characteristics of all included studies.

Name	Years	Country	Exercise type	Age	Tumor staging	Sample size	Intervention time	Intervention frequency	Exercise intensity	Outcomes
Gokal (42)	2016	UK	0E/CG	52.08 ± 11.7/ 52.36 ± 8.9	I-III	25/25	12weeks	5 times a week, >20min/times	NA	FACT-F/HADS-D
Pinto (43)	2005	USA	HE/CG	53.14 ± 9.70	0-II	43/43	12weeks	> 30 minutes, 5 days a week	moderate-intensity	Linear analog scale for fatigue)
Mock (44)	2005	USA	HE/CG	51.3± 8.9/ 51.6 ± 9.7	0–III	54/54	6weeks	> 60 minutes per week	moderate-intensity (50–70% maximum heart rate)	PFS
Husebø (45)	2014	Norway	OE/CG	50.8 ± 9.7/ 53.6 ± 8.8	I–III	33/34	15 weeks	30 minutes/day	moderate-intensity	SCFS-6
Mock (46)	2001	USA	0E/CG	48.64 ± 10.69/ 27.95 ± 5.94	I-IIIa	28/22	NA	> 90 minutes per week	NA	PFS
Wang (47)	2011	USA	0E/CG	48.40 ± 10.15/ 52.3± 8.84	I-II	30/32	6weeks	3-5 times a week	Low to moderate intensity(40–60% maximum heart rate)	FACT-F/PSQI
Han (48)	2019	China	TC/CG	46.39 ± 5.79/45.52 ± 6.50	I-III	23/21	12weeks	2 times/day, 5 days/week	NA	PFS-R
Yang (49)	2022	China	TC/CG	NA/NA	I-III	43/43	12weeks	20 minutes/time, 2 times/day, 5 days/week	NA	PFS-R/FACT-B
Hui (50)	2022	China	TC/CG	69.51 ± 5.73	I-III	49/49	6 months	2 times/day, 20 minutes/time	NA	CFS/PSQI/FACT-B
Xie (51)	2022	China	DE/OE	18~50	I-IV	45/45	Four cycles of chemotherapy	3 ~ 4 times/week, 30 ~ 40 min/time	NA	CFS
Xu (52)	2012	China	OE/CG	47.3±12.8	NA	39/39	8weeks	20- 30 minutes/time	moderate-intensity (55–65% maximum heart rate)	RPFS
Hao (53)	2013	China	HE/CG	46 ± 11.13/48 ± 11.32	I-III	28/28	15weeks	For the first 3 weeks, 3 times a week, 15min each time, then increase by 5min every 3 weeks, and reach 35min in 13-15 weeks.	moderate-intensity (50–70% maximum heart rate)	RPFS
Cohen (54)	2021	USA	ME/OE	59.71 ± 6.99/58.56 ± 10.41/ 53.62± 8.03	I-III	13/14/13	NA	90min, three times/week.	moderate-intensity (50–70% maximum heart rate)	PFS
Courneya (55)	2007	Canada	0E/RT/CG	49/49.5/49	I-III	78/82/82	17weeks	Three times/week	NA	FACT-F
Moadel (56)	2007	USA	YG/CG	55.11 ± 10.07/54.23 ± 9,81	I-IV	108/56	12weeks	12sessions/week, 90minutes/session	NA	FACIT-F
Bower (57)	2012	USA	YG/CG	54.4 ± 5.7/ 53.3 ± 4.9	0-II	16/15	12weeks	Twice/week, 90 minutes/time	NA	FSI/PSQI/BDI
Vadiraja (58)	2017	India	YG/CG	50.54± 8.53	NA	33/31	3 months	NA	NA	FSI
Vardar (59)	2015	Turkey	ME/0E	49.89 ± 4.65/47.38 ± 7.57	I-II	19/21	6weeks	30 minutes/day, 3 days/week	60–70% maximum heart rate	EORTC QOL-C30 -Fatigue/
Cramer (60)	2015	Germany	YG/CG	48.3 ± 4.8/ 50.0 ± 6.7	I-III	19/21	12weeks	90min/week	NA	FACIT-F/FACT -B/HADS-D
Vadiraja (61)	2009	India.	YG/CG	50.54± 8.53	II-III	44/44	6weeks	> 3 times/week	NA	EORTC QoL C30 Fatigue
Chaoul (62)	2018	USA	YG/SE/CG	49.5 ± 9.8/ 50.4 ± 10.3/ 49 ± 10.1	I–III	74/68/85	12weeks	75-90min/time	NA	BFI/PSQI
Lötzke (63)	2016	Germany	YG/CG	51.0 ± 11.0/ 51.4 ± 11.1	NA	45/47	12weeks	60 minutes/week	NA	EORTC QLQ-C30 Fatigue
Banasik (64)	2011	USA	YG/CG	63.33 ± 6.9/ 62.4 ± 7.3	II-IV	9/9	8weeks	90 minutes/time	NA	Fatigue Likert Scale
Strunk (65)	2018	Germany	OE/CG	54.2 ± 7.8/ 51.5 ± 8.4	NA	30/21	24weeks	Twice/week, 90 minutes/time	NA	QLQ-C30 Fatigue
Danhauer (66)	2009	USA	YG/CG	54.3 ± 9.6 /57.2 10.2	Ductal carcinoma *in situ* -IV	13/14	10weeks	75 minutes/10 weeks	NA	FACT-F/PSQI/FACT-B/CES-D
Jong (67)	2018	Netherlands	YG/CG	51 ± 8/ 51 ± 7.3	I-III	47/36	12weeks	Once a week	NA	EORTC QLQ-C30, Fatigue/CES-D
Wang (68)	2014	China	YG/CG	18~60	NA	40/42	4 months	4 times/week, 1 time/day, 50min/time	NA	CFS
Zeng (69)	2017	China	YG/CG	NA	NA	20/24/23/22	4 months	Once/two days, 30 min/once, once/two days, 40 min/once, NA, once/two days, 40 min/once	NA	CFS
Xiang (70)	2017	China	YG/CG	18~60	NA	20/22/24/23	16weeks	Once every 2 days, 30 min/every 2 days, 40min/every 2 days, 40min/NA each time	NA	CFS
Yang (71)	2020	China	ME/CG	49.17 ± 13.24/49.24 ± 12.09	NA	79/83	1 month	2-5 times/week	NA	PFS-R
Liu (72)	2018	China	OE/CG	55.2± 2.3/ 51.2± 3.2	NA	30/30	5weeks	The first 4 weeks, 15 min/day, from the fifth week after 30 min, > 3 times/week	NA	PFS-R
Li (73)	2019	China	OE/CG	48.0± 11.0/47.0 ± 10.0	NA	46/46	1weeks	NA	NA	PFS-R
Liu (74)	2015	China	OE/CG	18-62	NA	34/35	8weeks	15 min/day for the first 4 weeks, 30 min/day for the last 4 weeks, > 3 times/week.	NA	CRF/PSQI
Yu (75)	2020	China	HE/CG	44.01 ± 2.11/ 44.25 ± 2.24	NA	44/44	6weeks	30min/each time, 4 times/week,	NA	PFS-R
Chang (76)	2016	China	0E/CG	42.59 ± 6.37	I-IV	51/49	18weeks	ME: 2-3times/day,	NA	PSQI
Yu (77)	2021	China	YG/CG	38.8 ± 10.9/ 39.5 ± 10.3	II-III	59/59	8weeks	Twice/day, 3 ~ 5 times/week	NA	CFS
Yang (78)	2022	China	OE/CG	45. 56 ± 2. 37/ 45. 32 ± 2. 18	NA	32/32	During chemotherapy	30 min/day, > 3 times/week	NA	PFS-R/PSQI
Bolam (21)	2019	Sweden	RT/CG/OE	52.7 ± 10.3 /24.6 ± 4.8/ 24.8 ± 4.4	I–IIIa	74/60	6weeks	60 minutes/time, twice a week	NA	CRF
Wang (79)	2022	China	BE/CG	NA	NA	10/10	16weeks	3 times/week, 40min/each time	NA	PFS-R
Wei (80)	2022	China	BE/CG	40-75/ 40-75	I~III	35/35	12weeks	5 times/week, 30min/each time	NA	MFSI-SF/FACT-B/HADS
Schad (81)	2013	Germany	DE/CG	61.7± 9.4/ 59.3± 11.0	I-III	30/30	6weeks	One hour per week	NA	CFS/PSQI
Liao (82)	2022	China	BE/CG	53.12± 7.02/54.63± 8.44	I–III	33/35	12weeks	2 times/week, 90 minutes/week	NA	EORTC QLQ-C30-Fatigue/PSQI
Boing (83)	2017	Brazil	DE/CG	54.1 ± 7.6	NA	8/11	12weeks	twice a week, 60 minutes/time	NA	PFS-R/BDI
Naraphong (84)	2014	Thailand	ME/CG	46.36± 9.37/47.17± 6.87	I–IIIa	11/12	10weeks	3-5 days/week	NA	CRF
Chen (85)	2013	China	QGCG	45.3 ± 6.3/ 44.7 ± 9.7	0-III	49/46	3 months	40 minutes, 5 times a week	NA	BFI/PSQI/CES-D
Huang (86)	2016	China	TC/QC/CG	NA	NA	31/33	12 weeks	30 minutes/time	NA	EFS
Jiang (87)	2019	China	YG/CG	43.48 ± 9.72/42.63 ± 9.56	I-III	58/50	4weeks	2 times/day, 20min/each time	NA	PSQI/CFS
Rahmani (88)	2015	Iran	YG/CG	43.25± 3.07/44.08± 3.28	l-III	12/12	8weeks	Once/week, 2 hours/time	NA	FSS
Wang (89)	2017	China	TC/CG	50.5	I-III	45/41	3 months	20 minutes/time	NA	CFS/PSQI/SDS
Li (90)	2019	China	YG/CG	47.5 ± 8.2/46.7 ± 9.5	NA	45/45	2 months	60 min/time, 3 times/week	NA	PSQI
Xiong (91)	2019	China	0E/CG	51.8 ± 4.8/ 51.5 ± 4.6	NA	49/49	During chemotherapy	2 times/daily. 3 times/week	NA	PSQI/FACT-B
Zhuang (92)	2021	China	BE/CG	35-50	NA	70/70	12weeks	The first week 10min/time, 3 times/week, the second week extended to 10-20min/time, 4 times/week; Time in the third week 10-20min/time, 5 times/week.	moderate-intensity	PSQI/SDS
Stan (93)	2016	USA	YG/SE	61.4 ± 7.0/ 63.0± 9.3	0-II	18/16	12weeks	> 3 times/week	NA	FACT-B
Rogers (94)	2015	USA	HE/CG	54.9 ± 9.3/ 53.9 ± 7.7	I-III	105/112	3 months	> 3 times/week	moderate-intensity	FACT-B
Dieli (95)	2018	USA	ME/CG	NA	0-III	50/50	16weeks	> 3 times/week	moderate-intensity (65–85% maximum heart rate)	FACT-B/CES-D
Jin (96)	2017	China	YG/CG	55~73	NA	50/50	16weeks	3 times/week, 1 h/each time	NA	FACT-B/SDS
Du (97)	2019	China	0E/CG	50.1 ± 3.84/50.8 ± 3.00	I-III	40/40	4weeks	3 times/day, 20 minutes/time	55–65% maximum heart rate	FACT-B
Li (98)	2017	China	BE/CG	47.31 ± 9.85/45.43 ± 10.94	0-III	31/30	3 months	Once/day, 5day/week	NA	FACT-B/SDS
Odynets (99)	2019	Ukraine	OE/YG/PE	59.40 ± 1.24/59.10 ± 1.37	NA	45/30	12 months	3 times/week, 60 minutes/day	50–70% maximum heart rate	FACT-B
Fong (100)	2013	China	TC/CG	58.3 ± 10.1/53.8 ± 4.2	NA	12/16	6 months	3 times/week, 60 minutes/time	NA	FACT-B
Cai (101)	2022	China	YG/CG	45.62 ± 9.76/45.87 ± 9.43	I-IV	56/56	16Weeks	2 days/time,40 minutes/time	NA	SDS
Luo (102)	2021	China	BE/CG	48.5± 3.8/49.2 ± 3.2	I-III	35/35	4weeks	Once a week	NA	SDS
Leite (103)	2021	Brazil	DE/PE/CG	55.94± 10.98	0~III	18/18/16	16 weeks	Three times/week, 60 minutes/time	NA	BDI
Chen (104)	2021	China	OE/CG	41.45 ± 2.39/42.62 ± 1.73	NA	27/27	2 months	4 times/week, 20-30 minutes/time	56–65% maximum heart rate	CES-D
Liu (105)	2022	China	YG/CG	NA	1-II	61/62	NA	90min/week	NA	HADS-D

TC, Tai Chi; YG, Yoga; SE, Sling exercise; QG, Qigong; BE, Baduanjin exercise; RT, Resistance training; OE, Other exercise; CG, Control group; HE, Home Exercise; DE, Dance exercise; ME, Multimodal exercise; PE, Pilates exercise; NA, Not applicable.

### Outcomes

#### CRF

**Figure 3:**
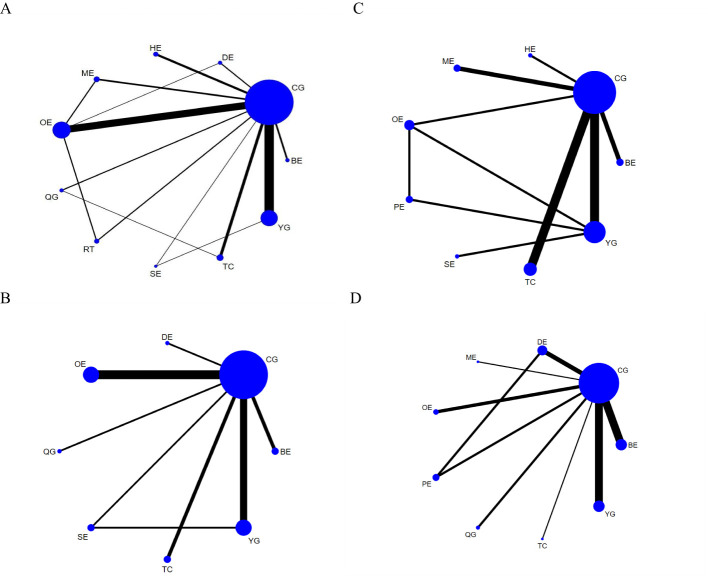
Network plots: **(A)** CRF, **(B)** Sleep quality, **(C)** Quality of life, **(D)** Depression. The size of the nodes represents the number of times the exercise appears in any comparison of that treatment, and the width of the edges represents the total sample size in the comparisons it connects. TC, Tai Chi; YG, Yoga; SE, Sling exercise; QG, Qigong; BE, Baduanjin exercise; RT, Resistance training; OE, Other exercise; CG, Control group; HE, Home Exercise; DE, Dance exercise; ME, Multimodal exercise; PE, Pilates exercise.

**Figure 4:**
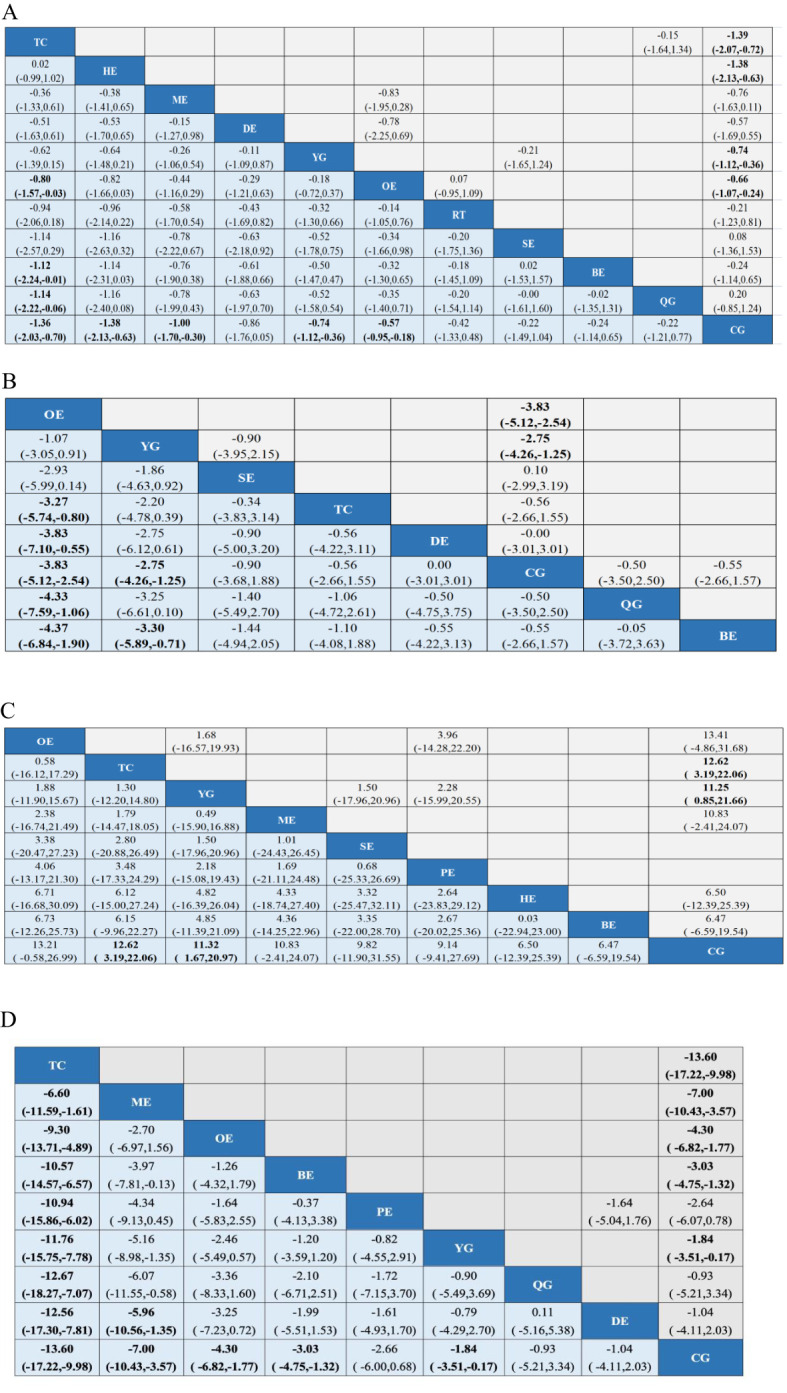
League tables of outcome analyses: **(A)** CRF, **(B)** Sleep quality, **(C)** Quality of life, **(D)** Depression. Data are mean differences and 95% credibility intervals for continuous data. TC, Tai Chi; YG, Yoga; SE, Sling exercise; QG, Qigong; BE, Baduanjin exercise; RT, Resistance training; OE, Other exercise; CG, Control group; HE, Home Exercise; DE, Dance exercise; ME, Multimodal exercise; PE, Pilates exercise.

Our study found that TC is significantly better than OE, BE, QG and CG in improving CRF in breast cancer patients. A total of 48 studies, involving 3766 participants, assessed CRF. In the NMA, 11 interventions were included (**Figure 3A**): Tai Chi (TC), Yoga (YG), Sling exercise (SE), Qigong (QG), Baduanjin exercise (BE), Resistance training (RT), Other exercise (OE), Control group (CG), Home Exercise (HE), Dance exercise (DE), Multimodal exercise (ME). Compared with OE, BE, QG, CG, TC improved significantly in CFR (SMD, -0.80; 95%CI, -1.57~-0.03), (SMD, -1.12; 95%CI, -2.24~-0.01), (SMD, -1.14; 95%CI, -2.22~-0.06), (SMD, -1.36; 95%CI, -2.03~-0.70) (**Figure 4A**). Comparison of adjusted funnel plots did not provide evidence of significant publication bias, as confirmed by Egger’s test (*P* = 0.030) (**Supplementary Material Appendix 5.1**). Heterogeneity, intransitivity, and inconsistencies in network meta-analyses were also evaluated (**Supplementary Material Appendix 6.1**). Furthermore, direct comparisons of the CRF were assessed. (**Supplementary Material Appendix 7.1**).

#### Sleep quality

Our study found that YG were significantly better than CG and BE in improving sleep quality in breast cancer patients. In 16 studies, PSQI was assessed in 1423 participants. 8interventions were included in the NMA (**Figure 3B**): Tai Chi (TC), Yoga (YG), Sling exercise (SE), Qigong (QG), Baduanjin exercise (BE), Other exercise (OE), Control group (CG), Dance exercise (DE). The effect of YG on improving sleep quality is better than CG (MD, -2.75; 95%CI, -4.26~-1.25) and BE(MD, -3.30; 95%CI, -5.89~-0.71) (**Figure 4B**). Comparison of the adjusted funnel plot did not provide evidence of significant publication bias, as confirmed by Egger’s test (*P* = 0.550) (**Supplementary Material Appendix 5.2**). Heterogeneity, inaccessibility, and inconsistencies in the network meta-analyses were evaluated (**Supplementary Material Appendix 6**). In addition, direct comparisons of the PSQI scores were evaluated. (**Supplementary Material Appendix 7.2**).

#### Quality of life

Our study found that TC and YG were significantly more effective than CG in improving the quality of life of breast cancer patients. A total of 14 studies evaluated FACT-B in 1114 participants. 9 interventions were included in the NMA (**Figure 3C**): Tai Chi (TC), Yoga (YG), Sling exercise (SE), Baduanjin exercise (BE), Other exercise (OE), Control group (CG), Home Exercise (HE), Multimodal exercise (ME), Pilates exercise (PE). Compared with CG, TC (MD,12.62; 95%CI, 3.19~22.06) and YG (MD,11.32; 95%CI, 1.67~20.97) are more beneficial to improve the quality of life of breast cancer patients (**Figure 4C**). The comparison of the adjusted funnel plots did not provide evidence of significant publication bias, as confirmed by Egger’s test (*P* = 0.074) (**Supplementary Material Appendix 5.3**). Heterogeneity, inaccessibility, and inconsistencies in network meta-analyses were also evaluated (**Supplementary Material Appendix 6**). In addition, direct comparisons of FACT-B were assessed (**Supplementary Material Appendix 7.3**).

#### Depression

Our study found that TC was significantly more effective than ME, OE, BE, PE, YG, QG, DE, CG in improving depression in breast cancer patients. A total of 18 studies evaluated depression in 1313 participants. 10 interventions were included in the NMA (**Figure 3D**): Tai Chi (TC), Yoga (YG), Qigong (QG), Dance exercise (DE), Baduanjin exercise (BE), Other exercise (OE), Control group (CG), Multimodal exercise (ME), Pilates exercise (PE). TC is better than ME (SMD,-6.60; 95%CI, -11.59~-1.61),OE(SMD,-9.30; 95%CI, -13.71~-4.89),BE(SMD,-10.57; 95%CI, -14.57~-6.57),PE(SMD,-10.94; 95%CI, -15.86~-6.02),YG(SMD,-11.76; 95%CI, -15.75~-7.78),QG(SMD,-12.67; 95%CI, -18.27~-7.07),DE(SMD,-12.56; 95%CI, -17.30~-7.81),CG(SMD,-13.60; 95%CI, -17.22~-9.98) in improving depression in breast cancer patients (**Figure 4D**). The comparison of the adjusted funnel plots did not provide evidence of significant publication bias, as confirmed by Egger’s test (*P* = 0.822) (**Supplementary Material Appendix 5.4**). Heterogeneity, inaccessibility, and inconsistencies in network meta-analyses were also evaluated (**Supplementary Material Appendix 6**). In addition, direct comparisons of depression were assessed (**Supplementary Material Appendix 7.4**).

## Discussion

Survivors of breast cancer frequently encounter various side effects, with CRF being particularly prevalent (106). CRF tends to persist and may lead to dysfunction, diminished quality of life, and the onset of negative emotions. Appropriate and well-designed exercise interventions play a vital role in mitigating physical discomfort, enhancing the immune system, addressing symptoms such as fatigue and insomnia, and managing psychological issues including anxiety and depression (107–109).

To further investigate the role of exercise in alleviating CRF and improving the overall quality of life in patients with breast cancer, a comprehensive study was conducted. This research involved an extensive literature review spanning from 2001 to 2022, identifying 65 relevant articles. The analysis evaluated 12 distinct interventions (Tai Chi, Yoga, Sling exercise, Qigong, Baduanjin exercise, Resistance training, Other exercise, Control group, Home Exercise, Dance exercise, Multimodal exercise, Pilates exercise) to determine which intervention most effectively ameliorates CRF, reduces depression, and enhances the quality of life in patients with breast cancer.

The results of this study suggest that TC demonstrates superior efficacy in mitigating CRF among patients with breast cancer compared to other forms of exercise (OE, BE, QG, CG). TC, a traditional Chinese practice, integrates body, mind, and spirit during physical activity. It comprises slow, fluid movements coordinated with diaphragmatic breathing, muscle stretching and relaxation, enhanced body awareness, and meditation. This comprehensive approach facilitates physical and mental equilibrium, enhancing patients’ stability and mobility. Several studies indicate that the diaphragmatic breathing technique employed in TC can modify breathing patterns, reduce respiratory rates, maintain airway patency for extended periods, engage respiratory muscles, improve cardiopulmonary function, and consequently alleviate fatigue (110, 111). Additionally, research has shown that TC can modulate immune function and reduce inflammation levels, contributing to fatigue reduction (112). Beyond its immunomodulatory effects, TC has been demonstrated to enhance neuromuscular responses, increase lower limb skeletal muscle strength, and improve muscle mass and bone density (113). TC may also promote vagus nerve regulation, reduce sympathetic nervous system activity, enhance mental well-being, and thereby mitigate fatigue (112). Some studies (114) have also associated CRF with insufficient physical exercise, which can lead to muscle atrophy, metabolic dysfunction, physical impairments, and a decline in cardiopulmonary function, resulting in fatigue. TC and QG share a common origin, both emphasizing the integration of mind and body, internal and external harmony, and promoting improved body function through guided movements (115). TC places greater emphasis on body control and flexible footwork, whereas fitness QG primarily involves stationary lower limb movements with a relatively limited range of motion (116). Fitness QG also focuses more on regulating qi and blood flow. These distinctions may explain why TC is more effective than fitness QG and BE in improving CRF in patients with breast cancer.

Patients with breast cancer demonstrate a significantly higher prevalence of sleep disturbances compared to those with other cancer types, with sleep disorders affecting over 60% of patients within two months after surgery (117). These disruptions can adversely affect the body’s immune, nervous, and endocrine systems, exacerbating symptoms such as fatigue, anxiety, depression, and other physiological dysfunctions. Consequently, this can compromise the effectiveness of cancer treatments, accelerate disease progression, diminish quality of life, and potentially increase the risk of tumor recurrence or metastasis (118, 119). Research indicates that persistent sleep disturbances are associated with increased complications and high mortality rates among patients with breast cancer (120–122). Thus, improving sleep quality in this population is of paramount importance. Our study reveals that yoga is more effective than CG and BE in enhancing sleep quality among patients with breast cancer. Yoga, a holistic mind–body therapy incorporating spiritual practice, physical exercise, controlled breathing, and meditation, has demonstrated effectiveness in inducing relaxation, mitigating fatigue, and significantly contributing to improved sleep quality (123, 124). The rhythmic movements of yoga promote overall muscle relaxation, reduce skeletal muscle tension, lower cortical arousal, and reduce oxygen consumption, thereby facilitating improved sleep patterns (125). Additionally, the prana (abdominal deep breathing) component of yoga enhances chest oxygenation, activates the parasympathetic nervous system, and fosters a calm physiological state, ultimately reducing psychological stress and promoting better sleep (126). Through meditation, individuals are guided to focus on their emotions and sensations, cultivating an accepting and serene mindset that further enhances sleep quality (127). Studies have shown that chemotherapy drugs (128), targeted drugs (129), endocrine drugs (130) and surgical methods (131) have specific impacts on the sleep quality of patients with breast cancer. Therefore, when aiming to improve sleep quality in this patient population, it is essential to consider not only the selection of exercise interventions but also other factors that influence sleep quality.

Our research indicates that TC is more effective than CG in alleviating depressive symptoms and improving the quality of life in patients with breast cancer. Depression, a common emotional disorder observed among these patients during diagnosis and treatment, significantly affects treatment outcomes, quality of life, and immune system function, including cellular and humoral immunity. It also increases the risk of breast cancer recurrence and metastasis, potentially shortening survival times and increasing cancer-related mortality rates (132, 133). Consequently, identifying effective exercise interventions to alleviate depression in patients with breast cancer is vital for improving treatment efficacy and reducing mortality. Our findings diverge from conventional meta-analyses (134), as we intentionally incorporated a larger pool of studies focusing specifically on depression in patients with breast cancer to enrich our analysis. After surgery or chemotherapy, many patients often experience altered self-perception and struggle to accept these changes, which can trigger negative physiological and psychological responses. These adverse emotional states can induce endocrine fluctuations, exacerbating depression and other emotional disturbances, all of which impede patients’ overall well-being and quality of life. Research suggests that TC reduces physiological arousal, promotes physical relaxation, reduces sympathetic nervous system activity, stimulates the release of mood-elevating endorphins and catecholamines, regulates emotional responses, encourages positive thinking, and ameliorates adverse psychological states in patients with breast cancer (135). Studies have demonstrated that both TC and BE can effectively improve depression (136, 137). However, our findings indicate that TC is more effective than BE in alleviating depression in patients with breast cancer. To our knowledge, there is a lack of direct evidence comparing the efficacy of TC and BE in addressing depression in this patient population. Future research should focus on direct studies examining the role of TC and BE in addressing depression among patients with breast cancer.

## Study strengths and limitations

This research has several notable strengths. Firstly, we employed network meta-analysis for direct and indirect comparisons of various interventions. Importantly, we meticulously categorized exercise interventions into 12 distinct types based on the characteristics of the exercises involved in each study, which may encompass one or multiple forms of exercise. Our investigation examines the impact of diverse intervention methods on CRF, Pittsburgh Sleep Quality Index scores, quality of life, and depression. The findings from this comprehensive study offer a valuable reference point for future research and clinical applications.

Despite the significant findings of this study, several limitations warrant consideration. First, the study did not account for the duration, intensity, or frequency of the interventions. Second, the quality of blinding in the included studies was suboptimal, and the outcome measurements primarily relied on subjective indicators, lacking objective parameters. Future research should incorporate objective measures for CRF, sleep quality, and depression, such as blood biochemical indicators or other biomarkers. Third, the inclusion of only English and Chinese literature may have introduced potential heterogeneity. Fourth, all studies featured small sample sizes, emphasizing the need for large-scale investigations in future research. Finally, this study did not consider the influence of tumor stage, treatment type, psychological state, and family circumstances, all of which could substantially influence the study results.

## Conclusion

Evidence from network meta-analyses strongly supports the effectiveness of TC in ameliorating CRF, reducing depressive symptoms, and enhancing the quality of life among patients with breast cancer. Furthermore, YG demonstrates potential to improve sleep quality in this patient population. However, the study’s findings are constrained by certain limitations. Future research on CRF in patients with breast cancer should incorporate larger sample sizes, validate results using objective measures (such as blood biochemical markers or other biomarkers), and identify appropriate exercise interventions tailored for these patients.

## Data Availability

The original contributions presented in the study are included in the article/**Supplementary Material**. Further inquiries can be directed to the corresponding author.

## References

[1] Siegel RL Miller KD Fuchs HE Jemal A . Cancer statistics, 2022. CA Cancer J Clin. (2022) 72:7–33. doi: 10.3322/caac.21708 35020204

[2] Avis NE Crawford S Manuel J . Quality of life among younger women with breast cancer. J Clin Oncol. (2005) 23:3322–30. doi: 10.1200/JCO.2005.05.130 15908646

[3] Bower JE Ganz PA Desmond KA Rowland JH Meyerowitz BE Belin TR . Fatigue in breast cancer survivors: occurrence, correlates, and impact on quality of life. J Clin Oncol. (2000) 18:743–53. doi: 10.1200/JCO.2000.18.4.743 10673515

[4] Ganz PA . Monitoring the physical health of cancer survivors: a survivorship-focused medical history. J Clin Oncol. (2006) 24:5105–11. doi: 10.1200/JCO.2006.06.0541 17093271

[5] Nowe E Friedrich M Leuteritz K Sender A Stöbel-Richter Y Schulte T . Cancer-related fatigue and associated factors in young adult cancer patients. J Adolesc Young Adult Oncol. (2019) 8:297–303. doi: 10.1089/jayao.2018.0091 30789284

[6] Xiaodong X Xiaoyu Z . Recent progress in cancer-related fatigue: Interpretation of NCCN(2018 edition) Guidelines for cancer-related fatigue. Chin Oncol clinic. (2018) 45:817–20. doi: 10.3969/j.issn.1000-8179.2018.16.676

[7] Cella D Davis K Breitbart W Curt G Fatigue Coalition . Cancer-related fatigue: prevalence of proposed diagnostic criteria in a United States sample of cancer survivors. J Clin Oncol. (2001) 19:3385–91. doi: 10.1200/JCO.2001.19.14.3385 11454886

[8] Phillips SM Mcauley E . Physical activity and fatigue in breast cancer survivors: a panel model examining the role of self-efficacy and depression. Cancer Epidemiol Biomarkers Prev. (2013) 22:773–81. doi: 10.1158/1055-9965.EPI-12-0983 PMC3650084 23456557

[9] Carlson MA Fradgley EA Bridge P Taylor J Morris S Coutts E . The dynamic relationship between cancer and employment-related financial toxicity: an in-depth qualitative study of 21 Australian cancer survivor experiences and preferences for support. Support Care Cancer. (2022) 30:3093–103. doi: 10.1007/s00520-021-06707-7 34850273

[10] Bøhn SH Vandraas KF Kiserud CE Dahl AA Thorsen L Ewertz M . Work status changes and associated factors in a nationwide sample of Norwegian long-term breast cancer survivors. J Cancer Surviv. (2024) 18(2):375–84. doi: 10.1007/s11764-022-01202-2 PMC10960762 35314959

[11] Tran TXM Jung SY Lee EG Cho H Cho J Lee E . Long-term trajectory of postoperative health-related quality of life in young breast cancer patients: a 15-year follow-up study. J Cancer Surviv. (2023) 17:1416–26. doi: 10.1007/s11764-022-01165-4 35279800

[12] Brown JC Huedo-Medina TB Pescatello LS Pescatello SM Ferrer RA Johnson BT . Efficacy of exercise interventions in modulating cancer-related fatigue among adult cancer survivors: a meta-analysis. Cancer Epidemiol Biomarkers Prev. (2011) 20:123–33. doi: 10.1158/1055-9965.EPI-10-0988 21051654

[13] Mishra SI Scherer RW Snyder C Geigle PM Berlanstein DR Topaloglu O . Exercise interventions on health-related quality of life for people with cancer during active treatment. Cochrane Database Syst Rev. (2012) 2012:Cd008465. doi: 10.1111/coa.2012.37.issue-5 22895974 PMC7389071

[14] Ruiz-Casado A Álvarez-Bustos A de Pedro CG Méndez-Otero M Romero-Elías M . Cancer-related fatigue in breast cancer survivors: A review. Clin Breast Cancer. (2021) 21:10–25. doi: 10.1016/j.clbc.2020.07.011 32819836

[15] Carayol M Bernard P Boiché J Riou F Mercier B Cousson-Gélie F . Psychological effect of exercise in women with breast cancer receiving adjuvant therapy: what is the optimal dose needed? Ann Oncol. (2013) 24:291–300. doi: 10.1093/annonc/mds342 23041586

[16] Rajarajeswaran P Vishnupriya R . Exercise in cancer. Indian J Med Paediatr Oncol. (2009) 30:61–70. doi: 10.4103/0971-5851.60050 20596305 PMC2885882

[17] Velthuis M Agasi-Idenburg SC Aufdemkampe G Wittink HM . The effect of physical exercise on cancer-related fatigue during cancer treatment: a meta-analysis of randomised controlled trials. Clin Oncol. (2010) 22:208–21. doi: 10.1016/j.clon.2009.12.005 20110159

[18] Mcneely ML Campbell KL Rowe BH Klassen TP Mackey JR Courneya KS . Effects of exercise on breast cancer patients and survivors: a systematic review and meta-analysis. CMAJ. (2006) 175:34–41. doi: 10.1503/cmaj.051073 16818906 PMC1482759

[19] Lin PJ Peppone LJ Janelsins MC Mohile SG Kamen CS Kleckner IR . Yoga for the management of cancer treatment-related toxicities. Curr Oncol Rep. (2018) 20:5. doi: 10.1007/s11912-018-0657-2 29388071 PMC5901971

[20] Serra MC Ryan AS Ortmeyer HK Addison O Goldberg AP . Resistance training reduces inflammation and fatigue and improves physical function in older breast cancer survivors. Menopause. (2018) 25:211–6. doi: 10.1097/GME.0000000000000969 PMC5771834 28832427

[21] Bolam KA Mijwel S Rundqvist H Wengström Y . Two-year follow-up of the OptiTrain randomised controlled exercise trial. Breast Cancer Res Treat. (2019) 175:637–48. doi: 10.1007/s10549-019-05204-0 PMC6534518 30915663

[22] Dong B Xie C Jing X Lin L Tian L . Yoga has a solid effect on cancer-related fatigue in patients with breast cancer: a meta-analysis. Breast Cancer Res Treat. (2019) 177:5–16. doi: 10.1007/s10549-019-05278-w 31127466

[23] Gray L Sindall P Pearson SJ . Does resistance training ameliorate cancer-related fatigue in cancer survivors? A systematic review with meta-analysis. Disabil Rehabil. (2024) 46(11):2213–22. doi: 10.1080/09638288.2023.2226408 37345506

[24] Olsson Möller U Beck I Rydén L Malmström M . A comprehensive approach to rehabilitation interventions following breast cancer treatment - a systematic review of systematic reviews. BMC Cancer. (2019) 19:472. doi: 10.1186/s12885-019-5648-7 31109309 PMC6528312

[25] Zou LY Yang L He XL Sun M Xu JJ . Effects of aerobic exercise on cancer-related fatigue in breast cancer patients receiving chemotherapy: a meta-analysis. Tumor Biol. (2014) 35:5659–67. doi: 10.1007/s13277-014-1749-8 24570186

[26] Zhou S Jlang J Zhang L Jiang T Huang S . Different exercise therapies in patients with cancer-related fatigue: A network meta-analysis. Nurs J Chin PLA. (2021) 38:65–68 + 88. doi: 10.3969/j.issn.1008-9993.2021.08.017

[27] Invernizzi M De Sire A Lippi L Venetis K Sajjadi E Gimigliano F . Impact of rehabilitation on breast cancer related fatigue: A pilot study. Front Oncol. (2020) 10:556718. doi: 10.3389/fonc.2020.556718 33194622 PMC7609789

[28] Penna F Ballarò R Costelli P . The redox balance: A target for interventions against muscle wasting in cancer cachexia? Antioxid Redox Signal. (2020) 33:542–58. doi: 10.1089/ars.2020.8041 32037856

[29] Idorn M Thor Straten P . Exercise and cancer: from “healthy” to “therapeutic”? Cancer Immunol Immunother. (2017) 66:667–71. doi: 10.1007/s00262-017-1985-z PMC5406418 28324125

[30] Bafeta A Trinquart L Seror R Ravaud P . Reporting of results from network meta-analyses: methodological systematic review. BMJ. (2014) 348:g1741. doi: 10.1136/bmj.g1741 24618053 PMC3949412

[31] Hoaglin DC Hawkins N Jansen JP Scott DA Itzler R Cappelleri JC . Conducting indirect-treatment-comparison and network-meta-analysis studies: report of the ISPOR Task Force on Indirect Treatment Comparisons Good Research Practices: part 2. Value Health. (2011) 14:429–37. doi: 10.1016/j.jval.2011.01.011 21669367

[32] Wu PY Huang KS Chen KM Chou CP Tu YK . Exercise, nutrition, and combined exercise and nutrition in older adults with sarcopenia: A systematic review and network meta-analysis. Maturitas. (2021) 145:38–48. doi: 10.1016/j.maturitas.2020.12.009 33541561

[33] Liu YC Hung TT Konara Mudiyanselage SP Wang CJ Lin MF . Beneficial exercises for cancer-related fatigue among women with breast cancer: A systematic review and network meta-analysis. Cancers (Basel). (2022) 15(1):151. doi: 10.3390/cancers15010151 36612147 PMC9817866

[34] Moher D Shamseer L Clarke M Ghersi D Liberati A Petticrew M . Preferred reporting items for systematic review and meta-analysis protocols (PRISMA-P) 2015 statement. Syst Rev. (2015) 4:1. doi: 10.1186/2046-4053-4-1 25554246 PMC4320440

[35] Kali A Srirangaraj S . EndNote as document manager for summative assessment. J Postgrad Med. (2016) 62:124–5. doi: 10.4103/0022-3859.174158 PMC4944344 26767973

[36] Meneses-Echávez JF González-Jiménez E Ramírez-Vélez R . Supervised exercise reduces cancer-related fatigue: a systematic review. J Physiother. (2015) 61:3–9. doi: 10.1016/j.jphys.2014.08.019 25511250

[37] Higgins JP Altman DG Gøtzsche PC Jüni P Moher D Oxman AD . The Cochrane Collaboration’s tool for assessing risk of bias in randomised trials. BMJ. (2011) 343:d5928. doi: 10.1136/bmj.d5928 22008217 PMC3196245

[38] Jardim PSJ Rose CJ Ames HM Echavez JFM Van de Velde S Muller AE . Automating risk of bias assessment in systematic reviews: a real-time mixed methods comparison of human researchers to a machine learning system. BMC Med Res Methodol. (2022) 22:167. doi: 10.1186/s12874-022-01649-y 35676632 PMC9174024

[39] Rücker GSG Krahn U König J . netmeta: Network meta-analysis with R. Available online at: https://cran.r-project.org/web/packages/netmeta/index.html.

[40] TEAM. RDC . A language and environment for statistical computing. Vienna: R Foundation for Statistical Computing. (2017). Available online at: http://www.R-project.org.

[41] Rücker G Schwarzer G . Ranking treatments in frequentist network meta-analysis works without resampling methods. BMC Med Res Methodol. (2015) 15:58. doi: 10.1186/s12874-015-0060-8 26227148 PMC4521472

[42] Gokal K Wallis D Ahmed S Boiangiu I Kancherla K Munir F . Effects of a self-managed home-based walking intervention on psychosocial health outcomes for breast cancer patients receiving chemotherapy: a randomised controlled trial. Support Care Cancer. (2016) 24:1139–66. doi: 10.1007/s00520-015-2884-5 26275768

[43] Pinto BM Frierson GM Rabin C Trunzo JJ Marcus BH . Home-based physical activity intervention for breast cancer patients. J Clin Oncol. (2005) 23:3577–87. doi: 10.1200/JCO.2005.03.080 15908668

[44] Mock V Frangakis C Davidson NE Ropka ME Pickett M Poniatowski B . Exercise manages fatigue during breast cancer treatment: a randomized controlled trial. Psychooncology. (2005) 14:464–77. doi: 10.1002/(ISSN)1099-1611 15484202

[45] Husebø AM Dyrstad SM Mjaaland I Søreide JA Bru E . Effects of scheduled exercise on cancer-related fatigue in women with early breast cancer. Sci World J. (2014) 2014:271828. doi: 10.1155/2014/271828 PMC3915861 24563628

[46] Mock V Pickett M Ropka ME Muscari Lin E Stewart KJ Rhodes VA . Fatigue and quality of life outcomes of exercise during cancer treatment. Cancer Pract. (2001) 9:119–27. doi: 10.1046/j.1523-5394.2001.009003119.x 11879296

[47] Wang YJ Boehmke M Wu YW Dickerson SS Fisher N . Effects of a 6-week walking program on Taiwanese women newly diagnosed with early-stage breast cancer. Cancer Nurs. (2011) 34:E1–13. doi: 10.1097/NCC.0b013e3181e4588d 20697267

[48] Qiong H Liu Y Shuangyan H Minghui Z Simin X Hui H . Study on the influence of eight forms of Taijiquan on cancer-induced fatigue in patients with breast cancer. J Guangxi Univ Chin Med. (2019) 22:30–4. doi: 10.3969/j.issn.2095-4441.2019.04.011

[49] Liu Y Qiong H Hui X Qiu X Wang L Chen L . Observation of curative effect of simple Taijiquan on cancer-induced fatigue in patients with breast cancer and influence of inflammatory factors. J Guizhou Univ Chin Med. (2022) 44:29–34. doi: 10.16588/j.cnki.issn2096-8426.2022.05.007

[50] Hui RU Zhou Z Jianfeng S . Effect observation of social support combined with Taijiquan exercise in elderly patients with breast cancer after surgery. Nurs Pract Res. (2022) 19:1268–72. doi: 10.3969/j.issn.1672-9676.2022.09.002

[51] Rong X Ruijun L Wenlin C Wan H . Effects of dance exercise therapy on cancer fatigue and nutritional status in young and middle-aged breast cancer patients during chemotherapy. Chin J Pract Nurs. (2022) 38:1074–9. doi: 10.3760/cma.j.cn211501-20210609-01636

[52] Ying X . Effect of home aerobic exercise on cancer-related fatigue in breast cancer outpatient patients undergoing chemotherapy. Hainan Med. (2012) 23:145–7. doi: 10.3969/j.issn.1003-6350.2012.19.064

[53] Nan H Quncao Y Xiaoyun K Libin G . Nursing study on the influence of aerobic exercise on cancer-induced fatigue in patients with breast cancer. Nurs Pract Res. (2013) 10:4–6. doi: 10.3969/j.issn.1672-9676.2013.12.002

[54] Cohen J Rogers WA Petruzzello S Trinh L Mullen SP . Acute effects of aerobic exercise and relaxation training on fatigue in breast cancer survivors: A feasibility trial. Psychooncology. (2021) 30:252–9. doi: 10.1002/pon.v30.2 33010183

[55] Courneya KS Segal RJ Mackey JR Gelmon K Reid RD Friedenreich CM . Effects of aerobic and resistance exercise in breast cancer patients receiving adjuvant chemotherapy: a multicenter randomized controlled trial. J Clin Oncol. (2007) 25:4396–404. doi: 10.1200/JCO.2006.08.2024 17785708

[56] Moadel AB Shah C Wylie-Rosett J Harris MS Patel SR Hall CB . Randomized controlled trial of yoga among a multiethnic sample of breast cancer patients: effects on quality of life. J Clin Oncol. (2007) 25:4387–95. doi: 10.1200/JCO.2006.06.6027 17785709

[57] Bower JE Garet D Sternlieb B Ganz PA Irwin MR Olmstead R . Yoga for persistent fatigue in breast cancer survivors: a randomized controlled trial. Cancer. (2012) 118:3766–75. doi: 10.1002/cncr.v118.15 PMC3601551 22180393

[58] Vadiraja HS Rao RM Nagarathna R Nagendra HR Patil S Diwakar RB . Effects of yoga in managing fatigue in breast cancer patients: A randomized controlled trial. Indian J Palliat Care. (2017) 23:247–52. doi: 10.4103/IJPC.IJPC_95_17 PMC5545948 28827926

[59] Vardar Yağli N Şener G Arikan H Sağlam M İnal İnce D Savcı S . Do yoga and aerobic exercise training have impact on functional capacity, fatigue, peripheral muscle strength, and quality of life in breast cancer survivors? Integr Cancer Ther. (2015) 14:125–32. 10.1177/1534735414565699 25567329

[60] Cramer H Rabsilber S Lauche R Kümmel S Dobos G . Yoga and meditation for menopausal symptoms in breast cancer survivors-A randomized controlled trial. Cancer. (2015) 121:2175–84. doi: 10.1002/cncr.v121.13 25739642

[61] Vadiraja SH Rao MR Nagendra RH Nagarathna R Rekha M Vanitha N . Effects of yoga on symptom management in breast cancer patients: A randomized controlled trial. Int J Yoga. (2009) 2:73–9. doi: 10.4103/0973-6131.60048 PMC2933732 20842268

[62] Chaoul A Milbury K Spelman A Basen-Engquist K Hall MH Wei Q . Randomized trial of Tibetan yoga in patients with breast cancer undergoing chemotherapy. Cancer. (2018) 124:36–45. doi: 10.1002/cncr.v124.1 28940301 PMC5735004

[63] Lötzke D Wiedemann F Rodrigues Recchia D Ostermann T Sattler D Ettl J . Iyengar-yoga compared to exercise as a therapeutic intervention during (Neo)adjuvant therapy in women with stage I-III breast cancer: health-related quality of life, mindfulness, spirituality, life satisfaction, and cancer-related fatigue. Evid Based Complement Alternat Med. (2016) 2016:5931816. 27019663 10.1155/2016/5931816 PMC4785257

[64] Banasik J Williams H Haberman M Blank SE Bendel R . Effect of Iyengar yoga practice on fatigue and diurnal salivary cortisol concentration in breast cancer survivors. J Am Acad Nurse Pract. (2011) 23:135–42. doi: 10.1111/j.1745-7599.2010.00573.x 21355946

[65] Strunk MA Zopf EM Steck J Hamacher S Hallek M Baumann FT . Effects of kyusho jitsu on physical activity-levels and quality of life in breast cancer patients. In Vivo. (2018) 32:819–24. doi: 10.21873/invivo.11313 PMC6117768 29936464

[66] Danhauer SC Mihalko SL Russell GB Campbell CR Felder L Daley K . Restorative yoga for women with breast cancer: findings from a randomized pilot study. Psychooncology. (2009) 18:360–8. doi: 10.1002/pon.v18:4 PMC3930083 19242916

[67] Jong MC Boers I Schouten van der Velden AP Meij SV Göker E Timmer-Bonte ANJH . A randomized study of yoga for fatigue and quality of life in women with breast cancer undergoing (Neo) adjuvant chemotherapy. J Altern Complement Med. (2018) 24:942–53. doi: 10.1089/acm.2018.0191 30247961

[68] Guofei W Shuhong W Pinglan J Zeng C . Intervention effect of yoga on cancer-induced fatigue in patients with breast cancer undergoing chemotherapy. J Cent South Univ (Medical). (2014) 39:1077–82. doi: 10.11817/j.issn.1672-7347.2014.10.016 25355261

[69] Jinfang Z . Effect of yoga combined with music relaxation training on cancer-induced fatigue in patients with breast cancer undergoing chemotherapy. Electronic J Pract Clin Nurs. (2017) 2:1–2. doi: 10.3969/j.issn.2096-2479.2017.19.001

[70] Dongyang X Mei W Hao W Liu J Liu J Cao Z . Effect of yoga combined with music relaxation training on cancer-induced fatigue in patients with breast cancer undergoing chemotherapy. Chin J Modern Nurs. (2017) 23:184–7. doi: 10.3760/cma.j.issn.1674-2907.2017.02.009

[71] Min Y Yanni D Jing X Wang X Li L Zheng J . Application effect of exercise intervention on cancer-induced fatigue in patients with breast cancer undergoing chemotherapy. Clin Med Res Pract. (2020) 5:161–3. doi: 10.19347/j.cnki.2096-1413.202031057

[72] Jiali L . Effect of exercise intervention on cancer-induced fatigue and sleep quality in patients with breast cancer undergoing chemotherapy. World J Sleep Med. (2018) 5:792–4. doi: 10.3969/j.issn.2095-7130.2018.07.013

[73] Xuanzhi L Huiying L Huizhen H Shen L . Effect of exercise intervention on cancer-induced fatigue and sleep quality in patients with breast cancer undergoing chemotherapy. World J Sleep Med. (2019) 6:1311–2. doi: 10.3969/j.issn.2095-7130.2019.09.057

[74] Lina L Lihua Z Hongxia F Sun X . Effect of exercise intervention on cancer-related fatigue and sleep quality in patients with breast cancer undergoing chemotherapy. Gen Nurs. (2015) 13:2190–1. doi: 10.3969/j.issn.1674-4748.2015.22.030

[75] Xin Y . Observation on the effect of aerobic exercise on cancer-induced fatigue in patients with breast cancer treated with radiotherapy. Chin Med Guide. (2020) 18:95–6. doi: 10.15912/j.cnki.gocm.2020.30.046

[76] Li C Jie Z Yan W Wang Y Li H Li X . Effect of music therapy combined with aerobic exercise on sleep quality in patients with chemotherapy after radical breast cancer surgery. Nurs Manage China. (2016) 16:989–94. doi: 10.3969/j.issn.1672-1756.2016.07.030

[77] Ximei Y . Effects of rehabilitation yoga exercises combined with emotional nursing on cancer-related fatigue in patients with breast cancer. Chin convalescent Med. (2021) 30:1185–9. doi: 10.13517/j.cnki.ccm.2021.11.020

[78] Li Y . To analyze the effects of exercise intervention on cancer-induced fatigue and sleep quality in patients with breast cancer undergoing chemotherapy. World J Sleep Med. (2022) 9:1414–6. doi: 10.3969/j.issn.2095-7130.2022.08.011

[79] Yanbing W Zhongguo C Li Z Liang W Zuo J Wang Z . Intervention effect of Baduanjin and pattern-doudou diabolo on breast cancer patients after surgery. J Hebei North Univ (Natural Sci Edition). (2022) 38:16–19 + 22. doi: 10.3969/j.issn.1673-1492.2022.02.005

[80] Wei X Yuan R Yang J Zheng W Jin Y Wang M . Effects of Baduanjin exercise on cognitive function and cancer-related symptoms in women with breast cancer receiving chemotherapy: a randomized controlled trial. Support Care Cancer. (2022) 30:6079–91. doi: 10.1007/s00520-022-07015-4 35416502

[81] SChad F Rieser T Becker S Groß J Matthes H Oei SL . Efficacy of tango argentino for cancer-associated fatigue and quality of life in breast cancer survivors: A randomized controlled trial. Cancers (Basel). (2023) 15(11):2920. doi: 10.3390/cancers15112920 37296883 PMC10251919

[82] Liao J Chen Y Cai L Wang K Wu S Wu L . Baduanjin’s impact on quality of life and sleep quality in breast cancer survivors receiving aromatase inhibitor therapy: a randomized controlled trial. Front Oncol. (2022) 12:807531. doi: 10.3389/fonc.2022.807531 35992855 PMC9388824

[83] Boing L Baptista F Pereira GS Sperandio FF Moratelli J Cardoso AA . Benefits of belly dance on quality of life, fatigue, and depressive symptoms in women with breast cancer - A pilot study of a non-randomised clinical trial. J Bodyw Mov Ther. (2018) 22:460–6. doi: 10.1016/j.jbmt.2017.10.003 29861250

[84] Naraphong W Lane A Schafer J Whitmer K Wilson BRA . Exercise intervention for fatigue-related symptoms in Thai women with breast cancer: A pilot study. Nurs Health Sci. (2015) 17:33–41. doi: 10.1111/nhs.2015.17.issue-1 24636322

[85] Chen Z Meng Z Milbury K Bei W Zhang Y Thornton B . Qigong improves quality of life in women undergoing radiotherapy for breast cancer: results of a randomized controlled trial. Cancer. (2013) 119:1690–8. doi: 10.1002/cncr.v119.9 PMC3852682 23355182

[86] Huang SM Tseng LM Chien LY Tai CJ Chen PH Hung CT . Effects of non-sporting and sporting qigong on frailty and quality of life among breast cancer patients receiving chemotherapy. Eur J Oncol Nurs. (2016) 21:257–65. doi: 10.1016/j.ejon.2015.10.012 26614591

[87] Zhen JJ Ping MX . Effects of mindfulness-based stress reduction training on perceived stress and cancer-related fatigue in patients with advanced breast cancer. Clin Educ Gen Pract. (2019) 17:575–6. doi: 10.13558/j.cnki.issn1672-3686.2019.03.033

[88] Rahmani S Talepasand S . The effect of group mindfulness - based stress reduction program and conscious yoga on the fatigue severity and global and specific life quality in women with breast cancer. Med J Islam Repub Iran. (2015) 29:175. 26034728 PMC4431452

[89] Yingying W . Effect of Taijiquan on cancer-related fatigue and quality of life in middle-aged and elderly patients with breast cancer after surgery. Anhui Normal University (2017).

[90] Jing L . Effects of yoga combined with meditation training on cancer-related fatigue and negative emotions in patients with breast cancer undergoing chemotherapy. Clin Nurs China. (2019) 11:284–7. doi: 10.3969/j.issn.1674-3768.2019.04.003

[91] Ping X . Application effect of music therapy combined with aerobic exercise in postoperative chemotherapy for breast cancer patients. Chin Contemp Med. (2019) 26:214–7. doi: 10.3969/j.issn.1674-4721.2019.14.064

[92] Ling ZY . Study of Baduanjin alleviating cancer-related fatigue in patients with breast cancer. Qingdao University (2021).

[93] Stan DL Croghan KA Croghan IT Jenkins SM Sutherland SJ Cheville AL . Randomized pilot trial of yoga versus strengthening exercises in breast cancer survivors with cancer-related fatigue. Support Care Cancer. (2016) 24:4005–15. doi: 10.1007/s00520-016-3233-z 27129840

[94] Rogers LQ Courneya KS Anton PM Hopkins-Price P Verhulst S Vicari SK . Effects of the BEAT Cancer physical activity behavior change intervention on physical activity, aerobic fitness, and quality of life in breast cancer survivors: a multicenter randomized controlled trial. Breast Cancer Res Treat. (2015) 149:109–19. doi: 10.1007/s10549-014-3216-z PMC4435784 25417174

[95] Dieli-Conwright CM Courneya KS Demark-Wahnefried W Sami N Lee K Sweeney FC . Aerobic and resistance exercise improves physical fitness, bone health, and quality of life in overweight and obese breast cancer survivors: a randomized controlled trial. Breast Cancer Res. (2018) 20:124. doi: 10.1186/s13058-018-1051-6 30340503 PMC6194749

[96] Cuifeng J Lili W Bei W . Effect of yoga exercise on cancer-induced fatigue and quality of life in breast cancer patients during chemotherapy. Integrated Nurs (Chinese Western Medicine). (2017) 3:12–5. doi: 10.11997/nitcwm.201704004

[97] Ping D Zheng Z Yao L Feng L . Clinical effect of aerobic exercise on oxygen carrying capacity and quality of life of breast cancer patients during chemotherapy. Rehabil China. (2019) 34:596–8. doi: 10.3870/zgkf.2019.11.010

[98] Qun L Fang WL Xin Z . Effect of Baduanjin on mood and quality of life of patients undergoing radiotherapy after radical breast cancer surgery. Gen Nurs. (2017) 15:2257–9. doi: 10.3969/j.issn.1674-4748.2017.18.033

[99] Odynets T Briskin Y Todorova V . Effects of different exercise interventions on quality of life in breast cancer patients: A randomized controlled trial. Integr Cancer Ther. (2019) 18:1534735419880598. doi: 10.1177/1534735419880598 31625419 PMC6801883

[100] Fong SS Ng SS Luk WS Chung JW Chung LM Tsang WW . Shoulder mobility, muscular strength, and quality of life in breast cancer survivors with and without tai chi qigong training. Evid Based Complement Alternat Med. (2013) 2013:787169. doi: 10.1155/2013/787169 23710237 PMC3655570

[101] Li C . Effect of music relaxation training combined with yoga exercise on breast cancer patients undergoing chemotherapy. Contemp Nurses (next issue). (2022) 29:142–5. doi: 10.19793/j.cnki.1006-6411.2022.12.042

[102] Yan L Shangzhong C Li S Jiying X Cuihua Z Ting P . Effects of Baduanjin combined with five elements music on anxiety and depression in breast cancer patients undergoing chemotherapy. Chin Community physician. (2021) 37:180–1. doi: 10.3969/j.issn.1671-6981.2007.02.04

[103] Leite B De Bem Fretta T Boing L Coutinho de Azevedo Guimarães A . Can belly dance and mat Pilates be effective for range of motion, self-esteem, and depressive symptoms of breast cancer women? Complement Ther Clin Pract. (2021) 45:101483. 34517217 10.1016/j.ctcp.2021.101483

[104] Ying C . Effects of home aerobic exercise on cancer-related fatigue and self-efficacy in breast cancer outpatient patients undergoing chemotherapy. Gen Nurs. (2021) 19:2522–5. doi: 10.12104/j.issn.1674-4748.2021.18.020

[105] Liu W Liu J Ma L Chen J . Effect of mindfulness yoga on anxiety and depression in early breast cancer patients received adjuvant chemotherapy: a randomized clinical trial. J Cancer Res Clin Oncol. (2022) 148:2549–60. doi: 10.1007/s00432-022-04167-y PMC9253261 35788727

[106] Berger AM Mooney K Alvarez-Perez A Breitbart WS Carpenter KM Cella D . Cancer-related fatigue, version 2.2015. J Natl Compr Canc Netw. (2015) 13:1012–39. doi: 10.6004/jnccn.2015.0122 PMC5499710 26285247

[107] Speck RM Courneya KS Mâsse LC Duval S Schmitz KH . An update of controlled physical activity trials in cancer survivors: a systematic review and meta-analysis. J Cancer Surviv. (2010) 4:87–100. doi: 10.1007/s11764-009-0110-5 20052559

[108] Gjerset GM Fosså SD Courneya KS Skovlund E Thorsen L . Exercise behavior in cancer survivors and associated factors. J Cancer Surviv. (2011) 5:35–43. doi: 10.1007/s11764-010-0148-4 20890674 PMC3040309

[109] Courneya KS . Physical activity in cancer survivors: a field in motion. Psychooncology. (2009) 18:337–42. doi: 10.1002/pon.v18:4 19306338

[110] Hong L Wujie H Zheng J . Research status of Taijiquan in postoperative rehabilitation of breast cancer patients. Chin J Rehabil Med. (2019) 34:984–8. doi: 10.3969/j.issn.1001-1242.2019.08.022

[111] Sitlinger A Brander DM Bartlett DB . Impact of exercise on the immune system and outcomes in hematologic Malignancies. Blood Adv. (2020) 4:1801–11. doi: 10.1182/bloodadvances.2019001317 PMC7189285 32343800

[112] Cheng D Wang X Hu J Dai LL Lv Y Feng H . Effect of tai chi and resistance training on cancer-related fatigue and quality of life in middle-aged and elderly cancer patients. Chin J Integr Med. (2021) 27:265–72. doi: 10.1007/s11655-021-3278-9 33420583

[113] Zhou W Wan YH Chen Q Qiu YR Luo XM . Effects of tai chi exercise on cancer-related fatigue in patients with nasopharyngeal carcinoma undergoing chemoradiotherapy: A randomized controlled trial. J Pain Symptom Manage. (2018) 55:737–44. doi: 10.1016/j.jpainsymman.2017.10.021 29122618

[114] Winters-Stone KM Bennett JA Nail L Schwartz A . Strength, physical activity, and age predict fatigue in older breast cancer survivors. Oncol Nurs Forum. (2008) 35:815–21. doi: 10.1188/08.ONF.815-821 18765328

[115] Yang H Yu D . Comparison of static balance function between Taijiquan exercise and fast walking exercise in middle-aged and elderly women. ChinJSports Med. (2013) 32:437–40. doi: 10.3969/j.issn.1000-6710.2013.05.010

[116] Yang H . Research on Exercise Preseription of Tai Chi for fitness in the elderly (doctoral thesis). Shanghai Sport University. (2011).

[117] Matthews EE Berger AM Schmiege SJ Cook PF McCarthy MS Moore CM . Cognitive behavioral therapy for insomnia outcomes in women after primary breast cancer treatment: a randomized, controlled trial. Oncol Nurs Forum. (2014) 41:241–53. doi: 10.1188/14.ONF.41-03AP 24650832

[118] Hanlon EC Van Cauter E . Quantification of sleep behavior and of its impact on the cross-talk between the brain and peripheral metabolism. Proc Natl Acad Sci U.S.A. (2011) 108 Suppl 3:15609–16. doi: 10.1073/pnas.1101338108 PMC3176603 21852576

[119] Chen D Yin Z Fang B . Measurements and status of sleep quality in patients with cancers. Support Care Cancer. (2018) 26:405–14. doi: 10.1007/s00520-017-3927-x 29058128

[120] Trudel-Fitzgerald C Zhou ES Poole EM Zhang X Michels KB Eliassen AH . Sleep and survival among women with breast cancer: 30 years of follow-up within the Nurses’ Health Study. Br J Cancer. (2017) 116:1239–46. doi: 10.1038/bjc.2017.85 PMC5418457 28359077

[121] Leysen L Lahousse A Nijs J Adriaenssens N Mairesse O Ivakhnov S . Prevalence and risk factors of sleep disturbances in breast cancersurvivors: systematic review and meta-analyses. Support Care Cancer. (2019) 27:4401–33. doi: 10.1007/s00520-019-04936-5 31346744

[122] Budhrani PH Lengacher CA Kip K Tofthagen C Jim H . An integrative review of subjective and objective measures of sleep disturbances in breast cancer survivors. Clin J Oncol Nurs. (2015) 19:185–91. doi: 10.1188/15.CJON.185-191 25840384

[123] Wang X Li P Pan C Dai L Wu Y Deng Y . The effect of mind-body therapies on insomnia: A systematic review and meta-analysis. Evid Based Complement Alternat Med. (2019) 2019:9359807. doi: 10.1155/2019/9359807 30894878 PMC6393899

[124] Kreutz C Schmidt ME Steindorf K . Effects of physical and mind-body exercise on sleep problems during and after breast cancer treatment: a systematic review and meta-analysis. Breast Cancer Res Treat. (2019) 176:1–15. doi: 10.1007/s10549-019-05217-9 30955185

[125] Daiqu Z Xiaojiang J Dengfen Z Yuan Z Ying L Yazhen L . Application of progressive relaxation training combined with acupressure in patients with chronic insomnia and mood disorders. Chongqing Med Sci. (2015) 44:3829–32. doi: 10.3969/j.issn.1671-8348.2015.27.028

[126] Turankar AV Jain S Patel SB Sinha SR Joshi AD Vallish BN . Effects of slow breathing exercise on cardiovascular functions, pulmonary functions & galvanic skin resistance in healthy human volunteers - a pilot study. Indian J Med Res. (2013) 137:916–21. PMC3734683 23760377

[127] Reich RR Lengacher CA Alinat CB Kip KE Paterson C Ramesar S . Mindfulness-based stress reduction in post-treatment breast cancer patients: immediate and sustained effects across multiple symptom clusters. J Pain Symptom Manage. (2017) 53:85–95. doi: 10.1016/j.jpainsymman.2016.08.005 27720794 PMC7771358

[128] Mills PJ Parker B Jones V Adler KA Perez CJ Johnson S . The effects of standard anthracycline-based chemotherapy on soluble ICAM-1 and vascular endothelial growth factor levels in breast cancer. Clin Cancer Res. (2004) 10:4998–5003. doi: 10.1158/1078-0432.CCR-0734-04 15297400

[129] Wittayanukorn S Qian J Johnson BS Hansen RA . Cardiotoxicity in targeted therapy for breast cancer: A study of the FDA adverse event reporting system (FAERS). J Oncol Pharm Pract. (2017) 23:93–102. doi: 10.1177/1078155215621150 26661047

[130] Desai K Mao JJ Su I Demichele A Li Q Xie SX . Prevalence and risk factors for insomnia among breast cancer patients on aromatase inhibitors. Support Care Cancer. (2013) 21:43–51. doi: 10.1007/s00520-012-1490-z 22584732 PMC3600410

[131] Miller AH Ancoli-Israel S Bower JE Capuron L Irwin MR . Neuroendocrine-immune mechanisms of behavioral comorbidities in patients with cancer. J Clin Oncol. (2008) 26:971–82. doi: 10.1200/JCO.2007.10.7805 PMC2770012 18281672

[132] Nakamura ZM Deal AM Nyrop KA Chen YT Quillen LJ Brenizer T . Serial assessment of depression and anxiety by patients and providers in women receiving chemotherapy for early breast cancer. Oncologist. (2021) 26:147–56. doi: 10.1002/onco.13528 PMC7873340 32946156

[133] Anuk D Özkan M Kizir A Özkan S . The characteristics and risk factors for common psychiatric disorders in patients with cancer seeking help for mental health. BMC Psychiatry. (2019) 19:269. doi: 10.1186/s12888-019-2251-z 31481035 PMC6724340

[134] Liu L Tan H Yu S Yin H Baxter GD . The effectiveness of tai chi in breast cancer patients: A systematic review and meta-analysis. Complement Ther Clin Pract. (2020) 38:101078. doi: 10.1016/j.ctcp.2019.101078 32056814

[135] Irwin MR Olmstead R Carrillo C Sadeghi N Nicassio P Ganz PA . Tai chi chih compared with cognitive behavioral therapy for the treatment of insomnia in survivors of breast cancer: A randomized, partially blinded, noninferiority trial. J Clin Oncol. (2017) 35:2656–65. doi: 10.1200/JCO.2016.71.0285 PMC5549450 28489508

[136] Yao LQ Kwok SWH Tan JB Wang T Liu XL Bressington D . The effect of an evidence-based Tai chi intervention on the fatigue-sleep disturbance-depression symptom cluster in breast cancer patients: A preliminary randomised controlled trial. Eur J Oncol Nurs. (2022) 61:102202. doi: 10.1016/j.ejon.2022.102202 36228406

[137] Luo X Zhao M Zhang Y Zhang Y . Effects of baduanjin exercise on blood glucose, depression and anxiety among patients with type II diabetes and emotional disorders: A meta-analysis. Complement Ther Clin Pract. (2023) 50:101702. doi: 10.1016/j.ctcp.2022.101702 36423358

