# Supplementary material for: Which specific modes of exercise training are most effective for breast related cancer fatigue? Network meta-analysis
Source: Front Oncol. 2025 Feb 26;15:1491634. doi: 10.3389/fonc.2025.1491634 (PMC11897559; doi:10.3389/fonc.2025.1491634)
Supplement: Supplementary file 1 [file DataSheet1.zip › Supplementary Material/Appendix 1-Search strategy.DOCX]

**Pubmed:**

| Number | Search terms | Results |
| --- | --- | --- |
| **#1** | ((((((((((((((((((((((((((((((((((((Breast Neoplasm[MeSH Terms]) OR (Neoplasm, Breast[Title/Abstract])) OR (Breast Tumors[Title/Abstract])) OR (Breast Tumor[Title/Abstract])) OR (Tumor, Breast[Title/Abstract])) OR (Tumors, Breast[Title/Abstract])) OR (Neoplasms, Breast[Title/Abstract])) OR (Breast Cancer[Title/Abstract])) OR (Cancer, Breast[Title/Abstract])) OR (Mammary Cancer[Title/Abstract])) OR (Cancer, Mammary[Title/Abstract])) OR (Cancers, Mammary[Title/Abstract])) OR (Mammary Cancers[Title/Abstract])) OR (Malignant Neoplasm of Breast[Title/Abstract])) OR (Breast Malignant Neoplasm[Title/Abstract])) OR (Breast Malignant Neoplasms[Title/Abstract])) OR (Malignant Tumor of Breast[Title/Abstract])) OR (Breast Malignant Tumor[Title/Abstract])) OR (Breast Malignant Tumors[Title/Abstract])) OR (Cancer of Breast[Title/Abstract])) OR (Cancer of the Breast[Title/Abstract])) OR (Mammary Carcinoma, Human[Title/Abstract])) OR (Carcinoma, Human Mammary[Title/Abstract])) OR (Carcinomas, Human Mammary[Title/Abstract])) OR (Human Mammary Carcinomas[Title/Abstract])) OR (Mammary Carcinomas, Human[Title/Abstract])) OR (Human Mammary Carcinoma[Title/Abstract])) OR (Mammary Neoplasms, Human[Title/Abstract])) OR (Human Mammary Neoplasm[Title/Abstract])) OR (Human Mammary Neoplasms[Title/Abstract])) OR (Neoplasm, Human Mammary[Title/Abstract])) OR (Neoplasms, Human Mammary[Title/Abstract])) OR (Mammary Neoplasm, Human[Title/Abstract])) OR (Breast Carcinoma[Title/Abstract])) OR (Breast Carcinomast[Title/Abstract])) OR (Carcinoma, Breast[Title/Abstract])) OR (Carcinomas, Breast[Title/Abstract]) | 467247 |
| **#2** | ((((((((((((((((((resistance training[Title/Abstract]) OR (swimming[Title/Abstract])) OR (running[Title/Abstract])) OR (jogging[Title/Abstract])) OR (walking[Title/Abstract])) OR(yoga[Title/Abstract])) OR (sports[Title/Abstract])) OR (cycling[Title/Abstract])) OR (physical activity[Title/Abstract])) OR (aerobics[Title/Abstract])) OR (TaiJi[Title/Abstract])) OR (QiGong[Title/Abstract])) OR (Exercise[Title/Abstract])) OR (yoga[Title/Abstract])) OR (Activity[Title/Abstract])) OR (Physical[Title/Abstract])) OR (Ba Duan Jin[Title/Abstract])) OR (Qigong[Title/Abstract])) OR (Dance[Title/Abstract]) | 4,350,356 |
| **#3** | #1 AND #2 | 67,169 |
| **#4** | ((fatigue[MeSH Terms]) OR (Lassitude[Title/Abstract])) OR (cancer-related fatigue[Title/Abstract]) | 39862 |
| **#5** | #3 AND #4 | 872 |

**Embase:**

| Number | Search terms | Results |
| --- | --- | --- |
| **#1** | 'breast neoplasm':ab,ti OR 'neoplasm, breast':ab,ti OR 'breast tumors':ab,ti OR 'breast tumor':ab,ti OR 'tumor, breast':ab,ti OR 'tumors, breast':ab,ti OR 'neoplasms, breast':ab,ti OR 'breast cancer':ab,ti OR 'cancer, breast':ab,ti OR 'mammary cancer':ab,ti OR 'cancer, mammary':ab,ti OR 'cancers, mammary':ab,ti OR 'mammary cancers':ab,ti OR 'malignant neoplasm of breast':ab,ti OR 'breast malignant neoplasm':ab,ti OR 'breast malignant neoplasms':ab,ti OR 'malignant tumor of breast':ab,ti OR 'breast malignant tumor':ab,ti OR 'breast malignant tumors':ab,ti OR 'cancer of breast':ab,ti OR 'cancer of the breast':ab,ti OR 'mammary carcinoma, human':ab,ti OR 'carcinoma, human mammary':ab,ti OR 'carcinomas, human mammary':ab,ti OR 'human mammary carcinomas':ab,ti OR 'mammary carcinomas, human':ab,ti OR 'human mammary carcinoma':ab,ti OR 'mammary neoplasms, human':ab,ti OR 'human mammary neoplasm':ab,ti OR 'human mammary neoplasms':ab,ti OR 'neoplasm, human mammary':ab,ti OR 'neoplasms, human mammary':ab,ti OR 'mammary neoplasm, human':ab,ti OR 'breast carcinoma':ab,ti OR 'breast carcinomas':ab,ti OR 'carcinoma, breast':ab,ti OR 'carcinomas, breast':ab,ti | 542009 |
| **#2** | 'resistance training':ab,ti OR swimming:ab,ti OR running:ab,ti OR jogging:ab,ti OR walking:ab,ti OR sports:ab,ti OR cycling:ab,ti OR 'physical activity':ab,ti OR aerobics:ab,ti OR taiji:ab,ti OR exercise:ab,ti OR yoga:ab,ti OR activity:ab,ti OR physical:ab,ti OR 'ba duan jin':ab,ti OR qigong:ab,ti OR dance:ab,ti OR pilates:ab,ti | 5462850 |
| **#3** | #1 AND #2 | 118309 |
| **#4** | 'fatigue'/exp OR fatigue OR 'lassitude'/exp OR lassitude OR 'cancer-related fatigue' OR ('cancer related' AND ('fatigue'/exp OR fatigue)) OR 'corticotropin releasing factor'/exp OR 'corticotropin releasing factor' | 121321 |
| **#5** | #3 AND #4 | 5233 |

**Web of science:**

| Number | Search terms | Results |
| --- | --- | --- |
| **#1** | TS=(Breast Neoplasm OR Neoplasm, Breast OR Breast Tumors OR Breast Tumor OR Tumor, Breast OR Tumors, Breast OR Neoplasms, Breast OR Breast Cancer OR Cancer, Breast OR Mammary Cancer OR Cancer, Mammary OR Cancers, Mammary OR Mammary Cancers OR Malignant Neoplasm of Breast OR Breast Malignant Neoplasm OR Breast Malignant Neoplasms OR Malignant Tumor of Breast OR Breast Malignant Tumor OR Breast Malignant Tumors OR Cancer of Breast OR Cancer of the Breast OR Mammary Carcinoma, Human OR Carcinoma, Human Mammary OR Carcinomas, Human Mammary OR Human Mammary Carcinomas OR Mammary Carcinomas, Human OR Human Mammary Carcinoma OR Mammary Neoplasms, Human OR Human Mammary Neoplasm OR Human Mammary Neoplasms OR Neoplasm, Human Mammary OR Neoplasms, Human Mammary OR Mammary Neoplasm, Human OR Breast Carcinoma OR Breast Carcinomas OR Carcinoma, Breast OR Carcinomas, Breast) | 750089 |
| **#2** | TS=(resistance training OR swimming OR running OR jogging OR walking OR yoga OR sports OR cycling OR physical activity OR aerobics OR TaiJi OR QiGong OR Exercise OR yoga OR Activity OR Physical OR Ba Duan Jin OR Qigong OR Dance OR Pilates) | 10,160,004 |
| **#3** | #1 AND #2 | 162051 |
| **#4** | TI=(fatigue OR Lassitude OR cancer-related fatigue OR CRF) | 132042 |
| **#5** | #3 AND #4 | 1017 |

**Cochrane:**

| Number | Search terms | Results |
| --- | --- | --- |
| **#1** | Breast Neoplasm or Neoplasm, Breast or Breast Tumors or Breast Tumor or Tumor, Breast or Tumors, Breast or Neoplasms, Breast or Breast Cancer or Cancer, Breast or Mammary Cancer or Cancer Mammary or Cancers, Mammary or Mammary Cancers or Malignant Neoplasm of Breast or Breast Malignant Neoplasm or Breast Malignant Neoplasms or Malignant Tumor of Breast or Breast Malignant Tumor or Breast Malignant Tumors or Cancer of Breast or Cancer of the Breast | 48341 |
| **#2** | resistance training OR swimming OR running OR jogging OR walking OR yoga OR sports OR cycling OR physical activity OR aerobics OR TaiJi OR QiGong OR Exercise OR yoga OR Activity OR Physical OR Ba Duan Jin OR Qigong OR Dance OR Pilates | 399610 |
| **#3** | #1 AND #2 | 9967 |
| **#4** | fatigue OR Lassitude OR cancer-related fatigue OR CRF | 53153 |
| **#5** | #3 AND #4 | 2552 |

**知网 (China National Knowledge Infrastructure , CNKI)**

| Number | Search terms | Results |
| --- | --- | --- |
| **#1** | 乳腺癌 + 乳腺肿瘤 + 乳房癌 + 乳癌 + 乳房肿瘤 |  |
| **#2** | 阻力训练 + 游泳 + 跑步 + 慢跑 + 散步 + 瑜伽 + 运动 + 自行车 + 体育活动 + 有氧运动 + 太极 + 气功 + 体育锻炼 + 瑜伽 + 活动 + 体育 + 八段锦 |  |
| **#3** | 疲乏 OR 癌因性疲乏 |  |
| **#4** | #1 AND #2 AND #3 | 149 |

**万方 (Wanfang)**

| Number | Search terms | Results |
| --- | --- | --- |
| **#1** | 乳腺癌 OR 乳腺肿瘤 OR 乳房癌 OR 乳癌 OR 乳房肿瘤 |  |
| **#2** | 阻力训练 OR 游泳 OR 跑步 OR 慢跑 OR 散步 OR 瑜伽 OR 运动 OR 自行车 OR 体育活动 OR 有氧运动 OR 太极 OR 气功 OR 体育锻炼 OR 瑜伽 OR 活动 OR 体育 OR 八段锦 |  |
| **#3** | 疲乏 OR 癌因性疲乏 |  |
| **#4** | #1 AND #2 AND #3 | 591 |

**维普(Chinese Medical Journal Database, VIP )**

| Number | Search terms | Results |
| --- | --- | --- |
| **#1** | 乳腺癌 OR 乳腺肿瘤 OR 乳房癌 OR 乳癌 OR 乳房肿瘤 |  |
| **#2** | 阻力训练 OR 游泳 OR 跑步 OR 慢跑 OR 散步 OR 瑜伽 OR 运动 OR 自行车 OR 体育活动 OR 有氧运动 OR 太极 OR 气功 OR 体育锻炼 OR 瑜伽 OR 活动 OR 体育 OR 八段锦 |  |
| **#3** | 疲乏 OR 癌因性疲乏 |  |
| **#4** | #1 AND #2 AND #3 | 78 |
