# Supplementary material for: Which specific modes of exercise training are most effective for breast related cancer fatigue? Network meta-analysis
Source: Front Oncol. 2025 Feb 26;15:1491634. doi: 10.3389/fonc.2025.1491634 (PMC11897559; doi:10.3389/fonc.2025.1491634)
Supplement: Supplementary file 1 [file DataSheet1.zip › Supplementary Material/Appendix 4-Risk of bias summary.pdf]

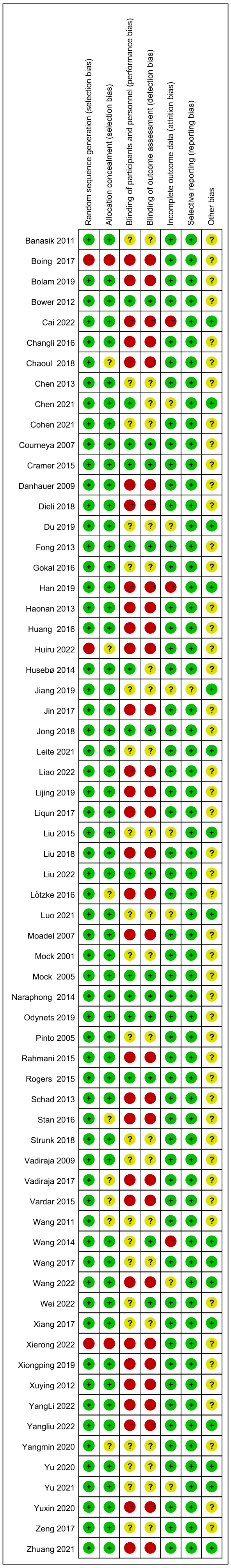
Random sequence generation (selection bias)
Allocation concealment (selection bias)
Blinding of participants and personnel (performance bias)
Blinding of outcome assessment (detection bias)
Incomplete outcome data (attrition bias)
Selective reporting (reporting bias)
Other bias
Banasik 2011
Boing 2017
Bolam 2019
Bower 2012
Cai 2022
Changli 2016
Chaoul 2018
Chen 2013
Chen 2021
Cohen 2021
Courneya 2007
Cramer 2015
Danhauer 2009
Dieli 2018
Du 2019
Fong 2013
Gokal 2016
Han 2019
Haonan 2013
Huang 2016
Huiru 2022
Husebø 2014
Jiang 2019
Jin 2017
Jong 2018
Leite 2021
Liao 2022
Lijing 2019
Liqun 2017
Liu 2015
Liu 2018
Liu 2022
Lötzke 2016
Luo 2021
Moadel 2007
Mock 2001
Mock 2005
Naraphong 2014
Odynets 2019
Pinto 2005
Rahmani 2015
Rogers 2015
Schad 2013
Stan 2016
Strunk 2018
Vadiraja 2009
Vadiraja 2017
Vardar 2015
Wang 2011
Wang 2014
Wang 2017
Wang 2022
Wei 2022
Xiang 2017
Xierong 2022
Xiongping 2019
Xuying 2012
YangLi 2022
Yangliu 2022
Yangmin 2020
Yu 2020
Yu 2021
Yuxin 2020
Zeng 2017
Zhuang 2021
