# Supplementary figures and images for: Which specific modes of exercise training are most effective for breast related cancer fatigue? Network meta-analysis
Source: Front Oncol. 2025 Feb 26;15:1491634. doi: 10.3389/fonc.2025.1491634 (PMC11897559; doi:10.3389/fonc.2025.1491634)

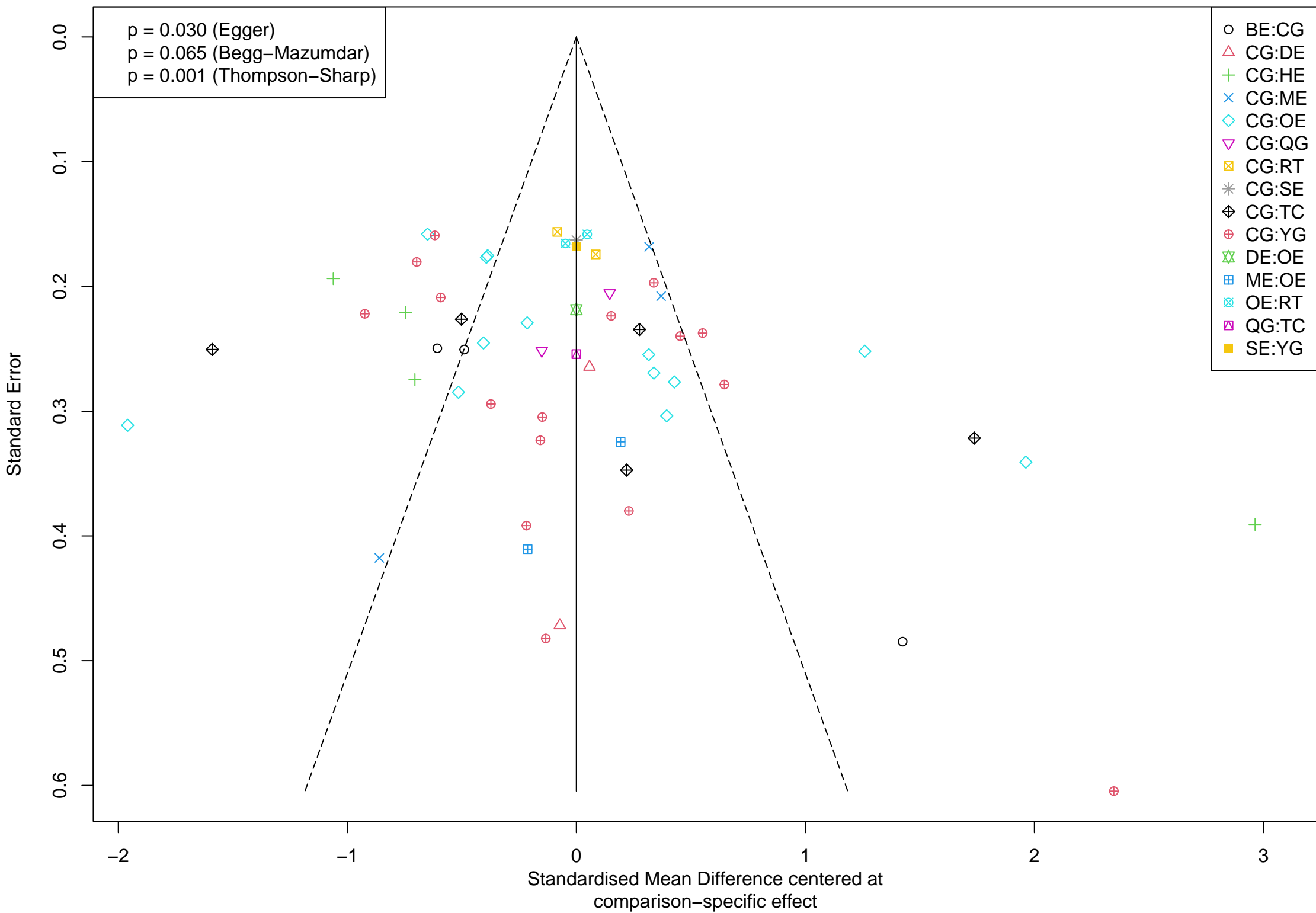

Supplement: Supplementary file 1 [file DataSheet1.zip › Supplementary Material/Appendix 5.1-Funnel polt.pdf]

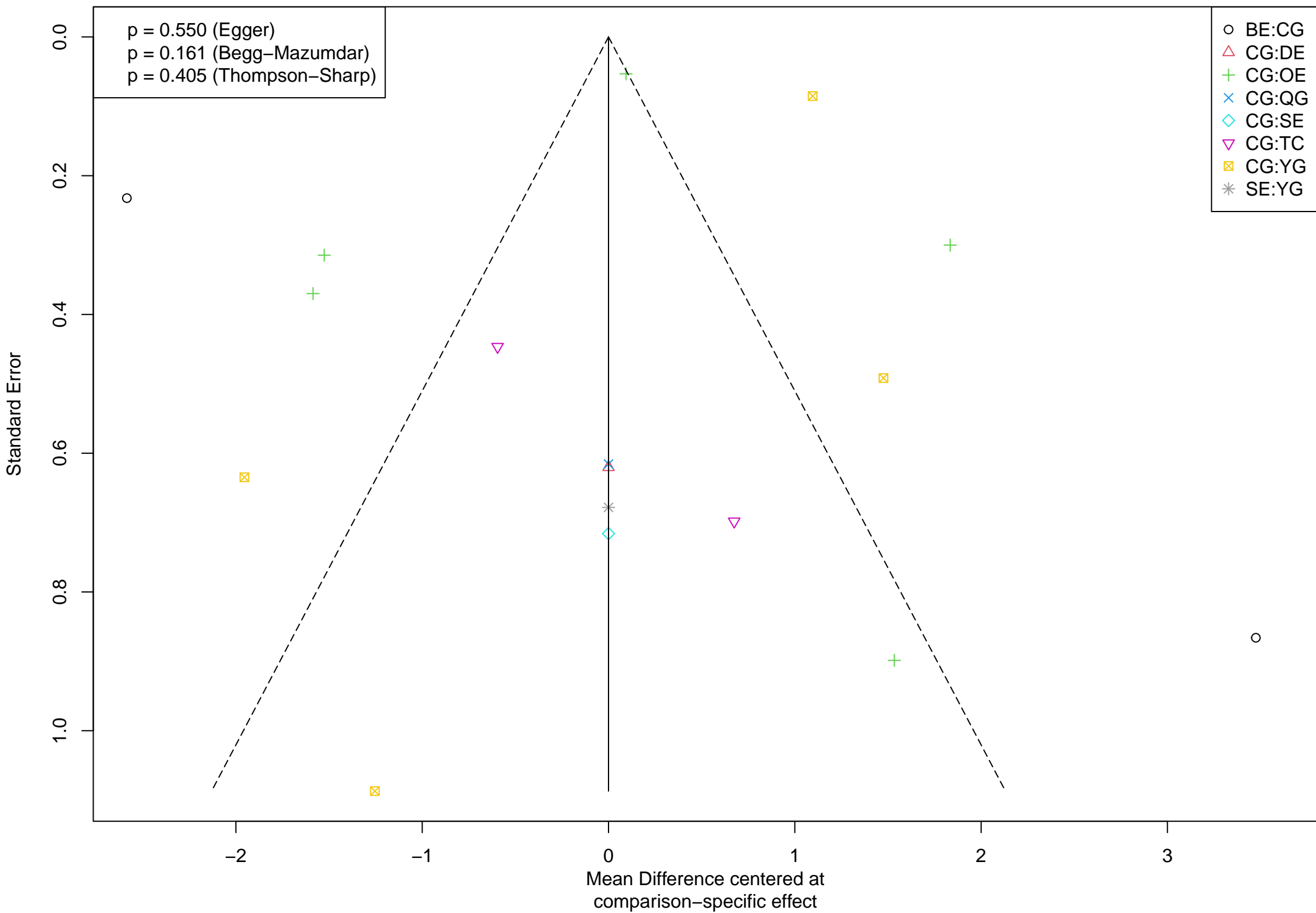

Supplement: Supplementary file 1 [file DataSheet1.zip › Supplementary Material/Appendix 5.2-Funnel polt.pdf]

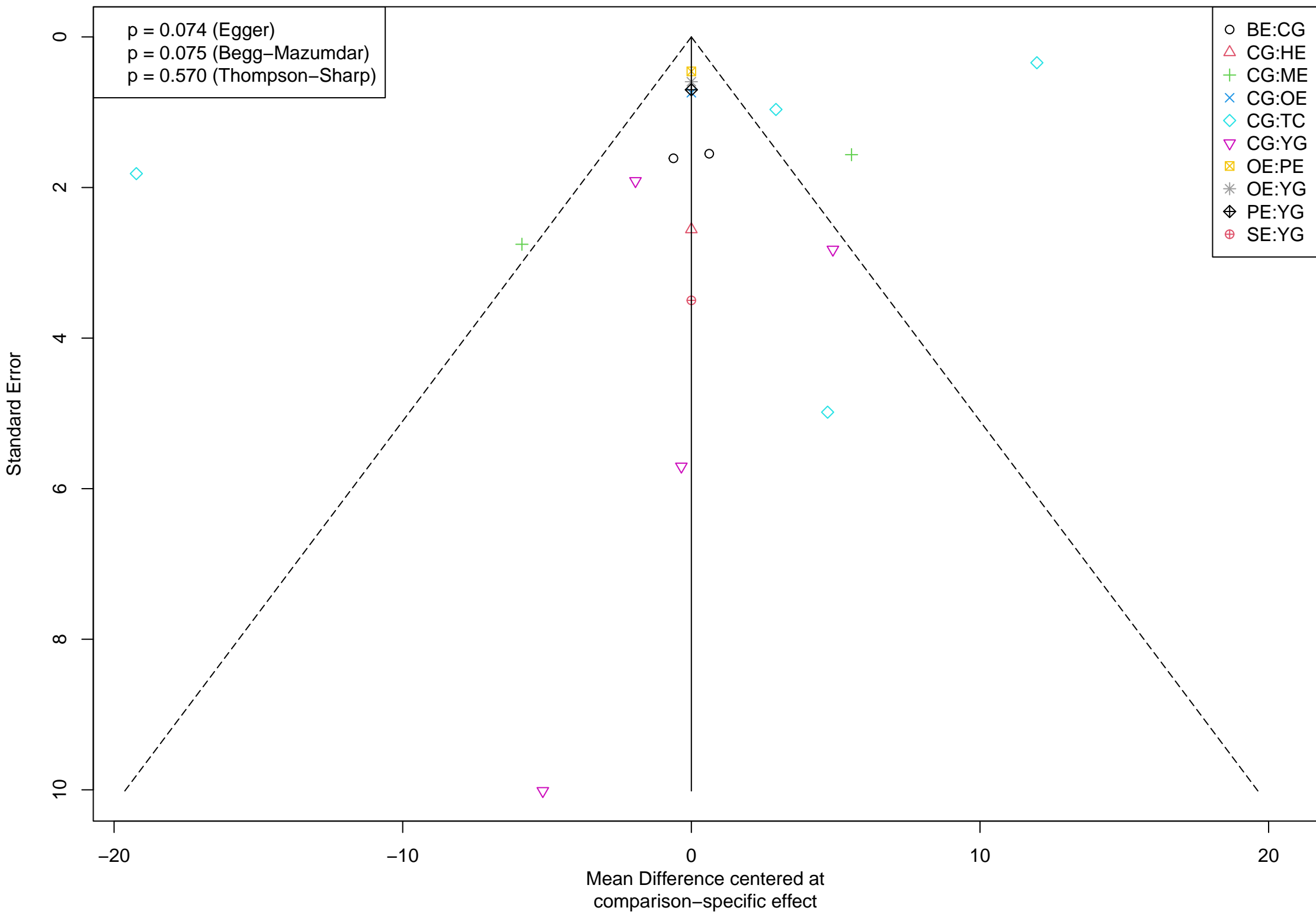

Supplement: Supplementary file 1 [file DataSheet1.zip › Supplementary Material/Appendix 5.3-Funnel polt.pdf]

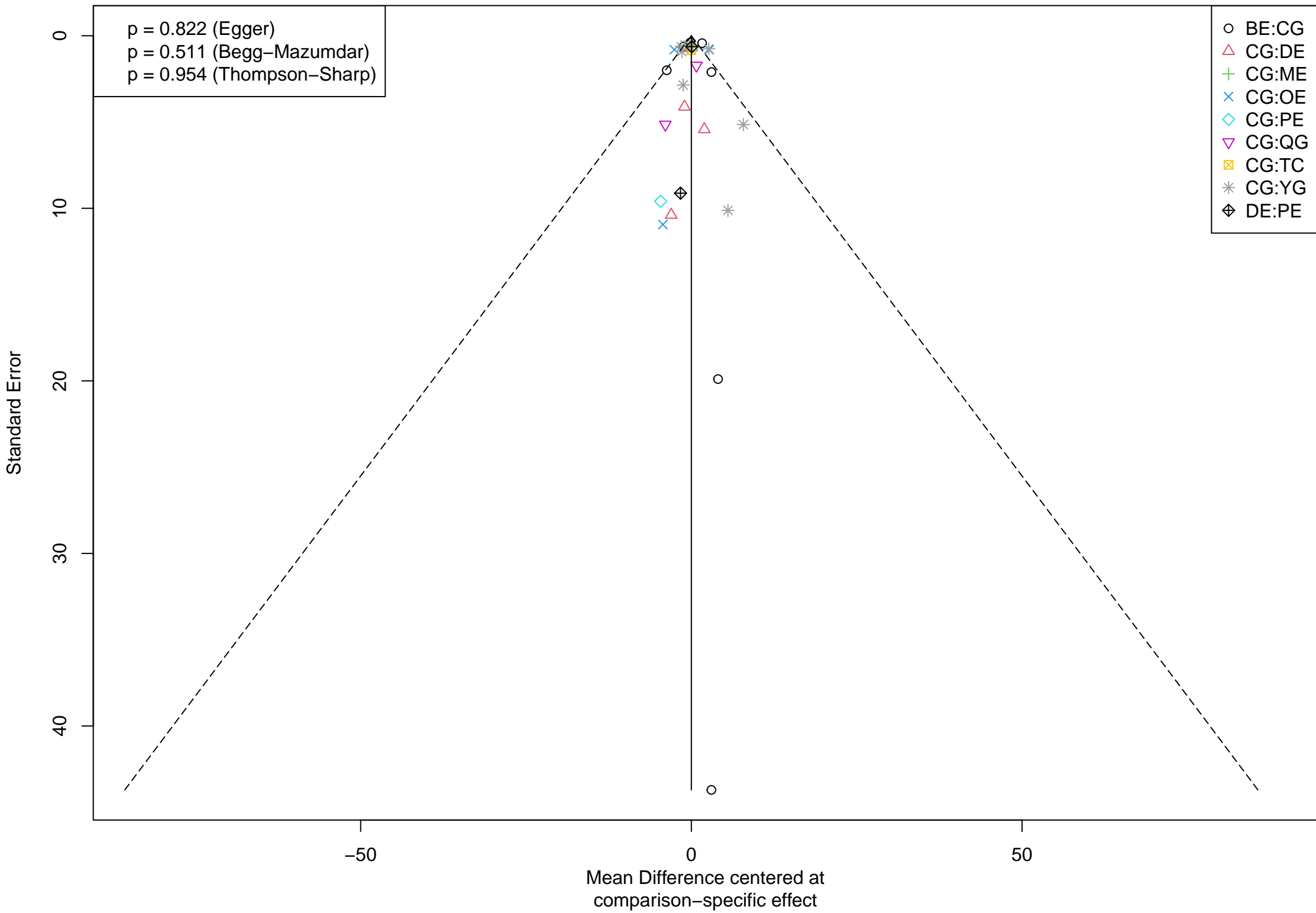

Supplement: Supplementary file 1 [file DataSheet1.zip › Supplementary Material/Appendix 5.4-Funnel polt.pdf]

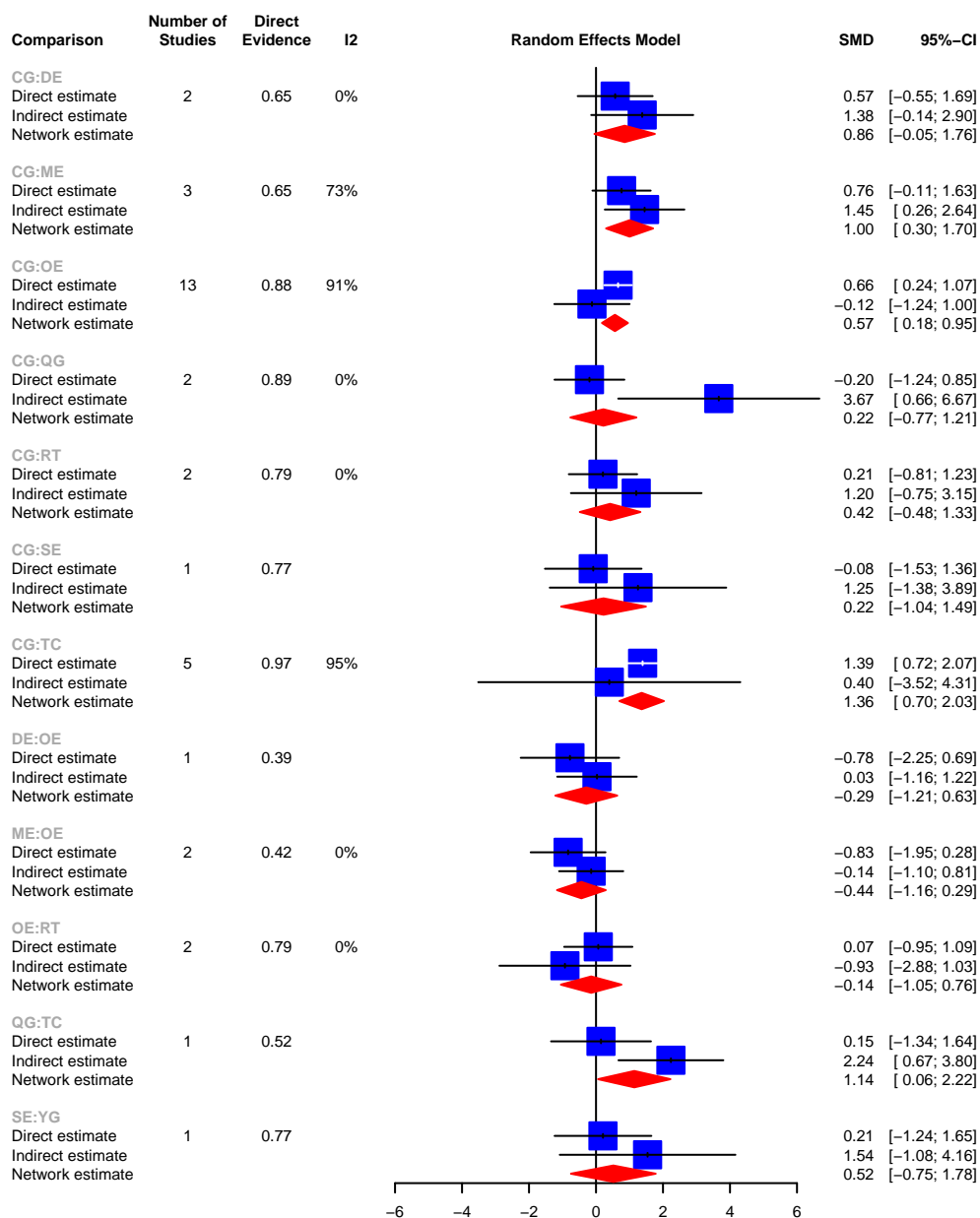

Supplement: Supplementary file 1 [file DataSheet1.zip › Supplementary Material/Appendix 7.1-Node split.pdf]

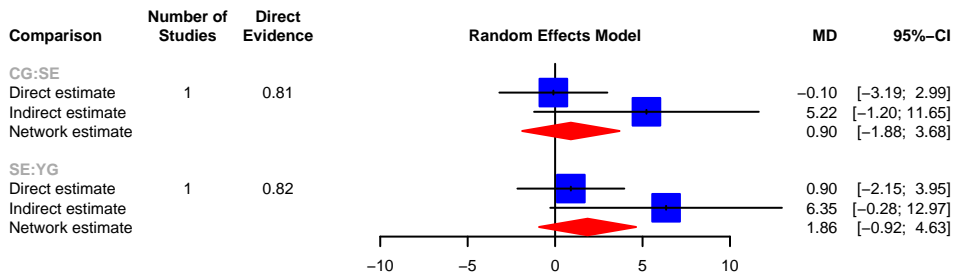

Supplement: Supplementary file 1 [file DataSheet1.zip › Supplementary Material/Appendix 7.2-Node split.pdf]

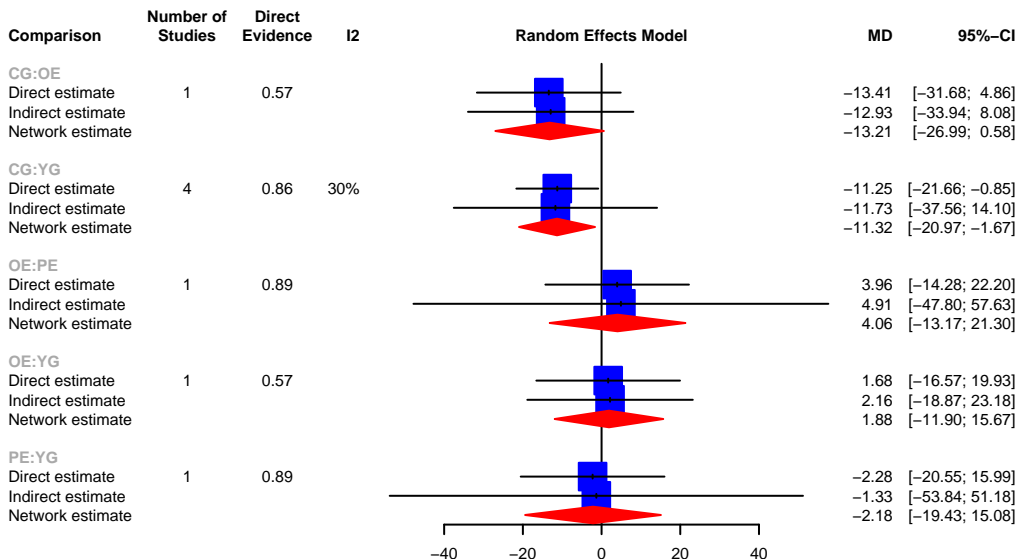

Supplement: Supplementary file 1 [file DataSheet1.zip › Supplementary Material/Appendix 7.3-Node split.pdf]

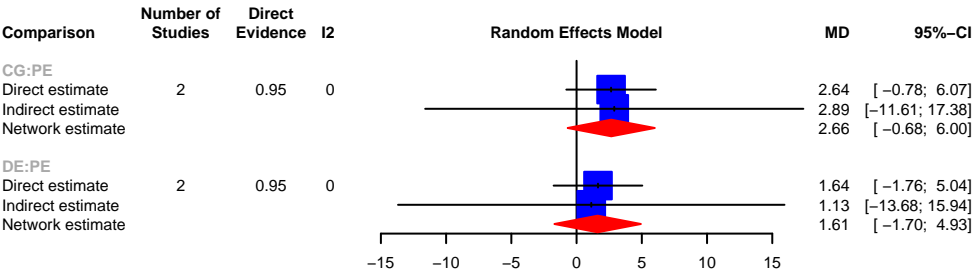

Supplement: Supplementary file 1 [file DataSheet1.zip › Supplementary Material/Appendix 7.4-Node split.pdf]
